# Mental health and psychosocial support interventions for populations affected by ongoing armed conflict: a scoping review

**DOI:** 10.1136/bmjgh-2025-022708

**Published:** 2026-05-27

**Authors:** Stéfanie Fréel, Janne L Punski-Hoogervorst, Chris M Hoeboer, Josef I Ruzek, Leisha Beardmore, Miranda Olff, Jana D Javakhishvili

**Affiliations:** 1 Department of Psychiatry, Amsterdam UMC Location University of Amsterdam, Amsterdam, The Netherlands; 2 Amsterdam Public Health, Global Health, Amsterdam, The Netherlands; 3 Deparment of Occupational Therapy, Faculty of Social Welfare and Health Sciences, University of Haifa, Haifa, Israel; 4 Palo Alto University, Palo Alto, California, USA; 5 Lyda Hill Institute of Human Resilience, University of Colorado Colorado Springs, Colorado Springs, Colorado, USA; 6 Department of Psychiatry and Behavioural Sciences, Stanford University School of Medicine, Stanford, California, USA; 7 ARQ National Psychotrauma Centre, Diemen, The Netherlands; 8 Institute of Addiction Studies, Faculty of Arts and Science, Ilia State University, Tbilisi, Georgia

**Keywords:** Mental Health & Psychiatry, Review, Treatment

## Abstract

**Introduction:**

Over 2 billion people worldwide are affected by ongoing armed conflict. The wide-ranging impact of conflict on populations’ mental health is well documented. However, less is known about the scope of mental health and psychosocial support (MHPSS) interventions implemented during periods of *active* armed conflict. This scoping review assesses the scope of published original articles and summarises existing evidence on MHPSS interventions for populations (civilians, internally displaced persons and veterans/ex-combatants) affected by ongoing armed conflict. It further outlines evidence gaps and proposed strategies to strengthen research and evaluation systems for MHPSS in these settings.

**Methods:**

The scoping review was conducted in accordance with the framework for scoping reviews described by Arksey and O’Malley (2005) and followed the guidelines for scoping reviews provided in the Preferred Reporting Items for Systematic reviews and Meta-Analyses, extension for Scoping Reviews (Tricco *et al*., 2018).

**Results:**

A total of 47 papers were included in the scoping review: 19 exclusively focused on children and adolescents, 14 exclusively on adults and 14 on both children/adolescents and adults. Across all population groups, only 12 papers investigated trauma-focused interventions. Many studies reported statistically significant positive outcomes in both adult civilians and ex-combatants with respect to decreased post-traumatic stress disorder, depression and anxiety symptomatology and improved psychosocial functioning, well-being and quality of life. Results were mixed in children/adolescents and when examining aggression, drug dependence and social/interpersonal skills in ex-combatants.

**Conclusion:**

The wide heterogeneity of research designs, results and clinical instruments used to assess changes in mental health and psychosocial outcomes across studies indicates the need for improved research standards when evaluating MHPSS interventions in ongoing conflict settings.

**WHAT IS ALREADY KNOWN ON THIS TOPIC**

- Mental health and psychosocial support (MHPSS) interventions have proven effective at both treating individual-level trauma-related conditions and addressing societal aspects of trauma in humanitarian settings. Significant evidence gaps however remain surrounding the scope, efficacy, feasibility and cultural relevance of psychological interventions mostly developed in high-income, western contexts when implemented specifically in *active* armed conflict settings.

**WHAT THIS STUDY ADDS**

- Overall, high study quality is lacking, and significant heterogeneity exists in intervention type, assessment scales, outcome targets and contextual details reported for MHPSS research conducted during active armed conflict. Evidence from this review indicates that various MHPSS interventions yielded statistically significant reductions in post-traumatic stress disorder (PTSD), depression and anxiety symptomatology, as well as improvements in psychosocial functioning, well-being and quality of life in both adult civilians and ex-combatants. Results were however mixed in children/adolescents and for interventions addressing aggression, drug dependence and social/interpersonal skills in adult ex-combatants.

**HOW THIS STUDY MIGHT AFFECT RESEARCH, PRACTICE OR POLICY**

- From a policy and programmatic standpoint, findings point to the need for a minimum set of MHPSS quality research standards and cultural adaptation protocols which balance intervention specificity through cultural and contextual attunement with generalisability for comparability, replicability and scale.

## Introduction

 Today, more countries are experiencing violent conflict than at any point since the Second World War, with 2 billion people affected worldwide.^1^ In 2024, the Uppsala Conflict Data Program (UCDP) recorded a total of 61 active state-based conflicts in which at least one of the parties involved was a government; 74 non-state conflicts occurring among ethnic, religious and other groups not involving government entities; and 49 one-sided perpetrations of violence whereby armed force or violent behaviour was used by the state government and/or non-state formally organized groups against civilians.^2^ In addition, 11 of the 61 active state-based conflicts reached the level of war—defined as conflicts with upwards of 1000 deaths per year, the majority occurring in Africa.^2^ In 2024, the deadliest wars occurred in Ukraine and Israel/Palestine.^2 3^ For the purpose of this paper, we define active armed conflict using an adaption of UCDP’s definition of the terms ‘active’ and ‘armed conflict’, namely “a conflict, both state-based and non-state, is deemed to be active if there are at least 25 battle-related deaths per calendar year […]” as a result of “the use of armed force between two parties”.^4 5^ Active armed conflict is further defined to include the timeframe following the recorded peak of violence within a given acute or ongoing conflict (e.g., during intermittent peace and/or reconciliation periods throughout a multiyear conflict) and settings not located in the direct line of active violence such as internally displaced persons (IDP)/refugee camps in a broader conflict affected region/country.

Conflict has wide-ranging and compounding effects on mental health. Individuals are often exposed to direct violence, forced displacement, persecution and the loss of livelihood, property and social networks—factors strongly associated with psychological distress and traumatic stress reactions.^6 7^ Conflict also damages social fabric and disrupts governance, econmic stability, and access to services,^8 9^ increasing exposure to insecurity and a wide range of human rights violations.^10^,^13^ An estimated 22% of people in conflict-affected settings develop mental disorders such as depression, anxiety, post-traumatic stress disorder (PTSD), bipolar disorder or schizophrenia,^14 15^ yet more than 80% of those in need do not receive care.^16 17^ Untreated mental health conditions can contribute to cycles of violence and instability^18^,^22^ and represent a global economic cost between US$2.5 and 8.5 trillion.^23^

The Inter-Agency Standing Committee (IASC) *Guidelines on Mental Health and Psychosocial Support in Emergency Settings* published in 2007 to coordinate multisectoral responses to humanitarian emergencies, including armed conflict, define mental health and psychosocial support (MHPSS) as “any type of local or outside support that aims to protect and promote psychosocial well-being and/or prevent or treat mental disorders” (IASC^24^, p. 16). MHPSS interventions span a wide spectrum—from clinical and preventive mental healthcare to community-based activities promoting social connection, safety, justice and resilience.^6 25^ While mental health is often linked to symptom-focused clinical care, psychosocial support typically involves non-specialist, community-level interventions aimed at enhancing well-being, social cohesion and access to basic services or livelihoods.^25^,^27^ Ongoing discussion continues around how these elements intersect, complement one another and interact with other factors—such as spiritual beliefs and practices,^28^ and social justice^29 30^—associated with healing, resilience and well-being within humanitarian practice.^30 31^

A systematic review and meta-analysis of 160 MHPSS activity reports identified a wide range of interventions commonly implemented in humanitarian settings.^6^ While evidence-based psychological interventions have proven effective in treating trauma-related disorders such as PTSD in emergency contexts, obstacles related to delivery persist.^32^,^36^ Significant challenges also remain in MHPSS research and implementation in conflict settings, specifically surrounding evidence-based psychological interventions aimed at addressing stress and stress-related disorders in contexts of *active* armed conflict.^37^ This includes evidence surrounding the types, quality and implementation modalities of such interventions as well as their efficacy, feasibility and cultural relevance, particularly when developed in high-income, western contexts and implemented in active armed conflict settings.^38^,^40^

Previous systematic reviews and meta-analyses have typically focused on assessing intervention effectiveness—rather than scope, among either highly specific populations such as women and children,^41 42^ refugees and asylum seekers,^43^ or following exposure to distinct types of traumatic events including natural disasters or sexual and gender-based violence^44^ within armed conflict settings. Other reviews of MHPSS interventions in humanitarian or emergency settings—while comprehensive, neither exclusively concentrate on populations exposed to *armed conflict* nor on an isolated conflict stage (eg, 645,47). Fewer solely focus on ongoing or *active* armed conflict settings, with many reviews including a large number of studies implemented in post-conflict settings—a gap which the current review seeks to address.^41 45 48^

In light of the enduring impact of armed conflict on mental health and the priorities outlined in the IASC consensus-based agenda for MHPSS (2021–2030),^49^ this paper seeks to answer the following research question: *What is the scope of the existing literature about mental health and psychosocial support interventions for populations affected by ongoing armed conflict and war since the post-Cold War period*? Specifically, it aims to (1) assess the scope of published original articles and summarise existing evidence on MHPSS interventions for populations affected by *ongoing* armed conflict and war since the post-Cold War period (ie, since the dissolution of the Soviet Union in 1991) and (2) identify evidence gaps and propose strategies to strengthen research, monitoring and evaluation systems for MHPSS in these settings.

## Methods

The scoping review was conducted in accordance with the framework for scoping reviews described by Arksey and O’Malley.^50^ This framework identifies five steps for the execution of scoping reviews, namely (1) identification of the research questions, (2) execution of search strategy, (3) selection of studies for inclusion, (4) extraction of data and (5) collating and reporting results. This review additionally followed the guidelines for scoping reviews as provided in the Preferred Reporting Items for Systematic reviews and Meta-Analyses, extension for Scoping Reviews.^51^ Finally, to contribute to open science and research transparency, the scoping review research protocol was pre-registered at the Open Science Framework (on 12 February 2024; osf.io/csr3t).

### Inclusion and exclusion criteria

The population of interest for this research was defined as any population living in an *active* armed conflict setting and undergoing a mental health and psychosocial intervention delivered within this context. Comparators included active and passive controls, treatment as usual, alternative treatment for randomised or quasi-randomised studies or no treatment for observational studies. Outcomes were defined to include trauma-related disorders and symptoms and general mental health status and psychosocial functioning. These elements were purposefully left broad to reflect (1) the diversity of MHPSS interventions captured in the literature across geographic locations affected by active armed conflict and (2) ongoing debate surrounding conceptual frameworks for MHPSS and the public health relevance of PTSD in humanitarian contexts (see ^6 30 52 53^).

Exclusion criteria included the intervention being implemented among populations located outside of active armed conflict settings—notably IDP or refugee camps located in countries outside active armed conflict zones. Other exclusion criteria involved studies conducted in both armed conflict and non-armed conflict settings and that did not clearly distinguish between these, studies only qualitatively reporting on the experiences of working with certain interventions, and case reports.

### Execution of the search strategy

A search strategy was developed based on the two pillars of this scoping review, namely (1) armed conflict (‘‘armed conflict’’ OR ‘‘war’’) and (2) mental health and psychosocial interventions (‘‘mental health and psychosocial support’’ OR ‘‘psychosocial support’’ OR ‘‘MHPSS’’ OR ‘‘psychological first aid’’ OR ‘‘mental health intervention’’ OR ‘‘mental health treatment’’ OR ‘‘mental health therapy’’ OR ‘‘psychological intervention’’ OR ‘‘psychological treatment’’ OR ‘‘psychological therapy’’).

To test the developed search strategy and assess the quality of the literature, an initial search strategy was conducted (by JLP-H) on 6 June 2023, in PsycINFO. The search identified 1373 articles, of which 25 articles were found suitable for the scoping review. Thus, it was decided to continue with the search strategy without making any adjustments. The full search was then executed (by JLP-H) on 5 May 2025 in EMBASE, PsycINFO, PubMed and Scopus. The results per database and the subsequent selection steps were imported into reference management software ‘Zotero’ and verified by a second researcher (SF or LB).

### Selection of studies for inclusion

The search was limited to English-language quantitative studies published in academic journals between January 1991 and May 2025. The start of the inclusion period was chosen as 1991 to include only studies conducted after the Cold War. The decision to focus on studies from the post-Cold War period was informed by shifts in the nature and proliferation of armed conflict,^54^ the institutionalisation of MHPSS practices and the availability of higher-quality and systematically documented interventions. This timeframe thus ensures relevance to contemporary humanitarian and clinical contexts.

### Data extraction

Removal of duplicates was done using the Zotero identification of duplicates function, with manual acceptance of deduplication to ensure accuracy. Data from the included studies were extracted and charted using a Microsoft Excel spreadsheet that was shared between reviewers (SF, JLP-H, CMH). The information to be extracted was decided upon a priori (ie, before the extraction of the data but after selection of relevant articles). The extracted data were as follows: (1) author and year of publication; (2) time and place of study conductance (ie, region and country); (3) status and duration of conflict (eg, acute or protracted conflict); (4) study design (eg, randomised controlled trial (RCT), presence of randomisation); (5) study population (eg, child, adolescent or adult; civilians, internally displaced persons, refugees or ex-combatants), number of participants, gender and age; (6) intervention details (eg, type, delivery modality and setting); (7) intervention provider and supervision; (8) main outcomes/effectiveness of the intervention and (9) primary target of the intervention. Details of each study were extracted by one of the reviewers (SF, JLP-H, CMH) and verified by the others. Any difficulties with data extraction were discussed during online discussion meetings.

### Collating and reporting results

Following data extraction, entries from the included studies were reviewed and discussed between reviewers (SF, JLP-H, CMH). If two reviewers disagreed on relevance of a manuscript, the third reviewer was consulted. Given that many included articles did not report the specific timeframe of the conducted research, this information was searched separately by the reviewers (SF, JLP-H, CMH) through both a Google search and cross-reference to the UCDP database.^55^ Extracted data were collated and reported per study population, namely i) children and adolescents, ii) children, adolescents and adults, and adults only. Within each of these, other extracted data were reported under the following categories: conflict-specific information including region, timeframe and context, study population, intervention type and design, intervention providers and supervision, and intervention outcomes.

## Results

**Figure 1:**
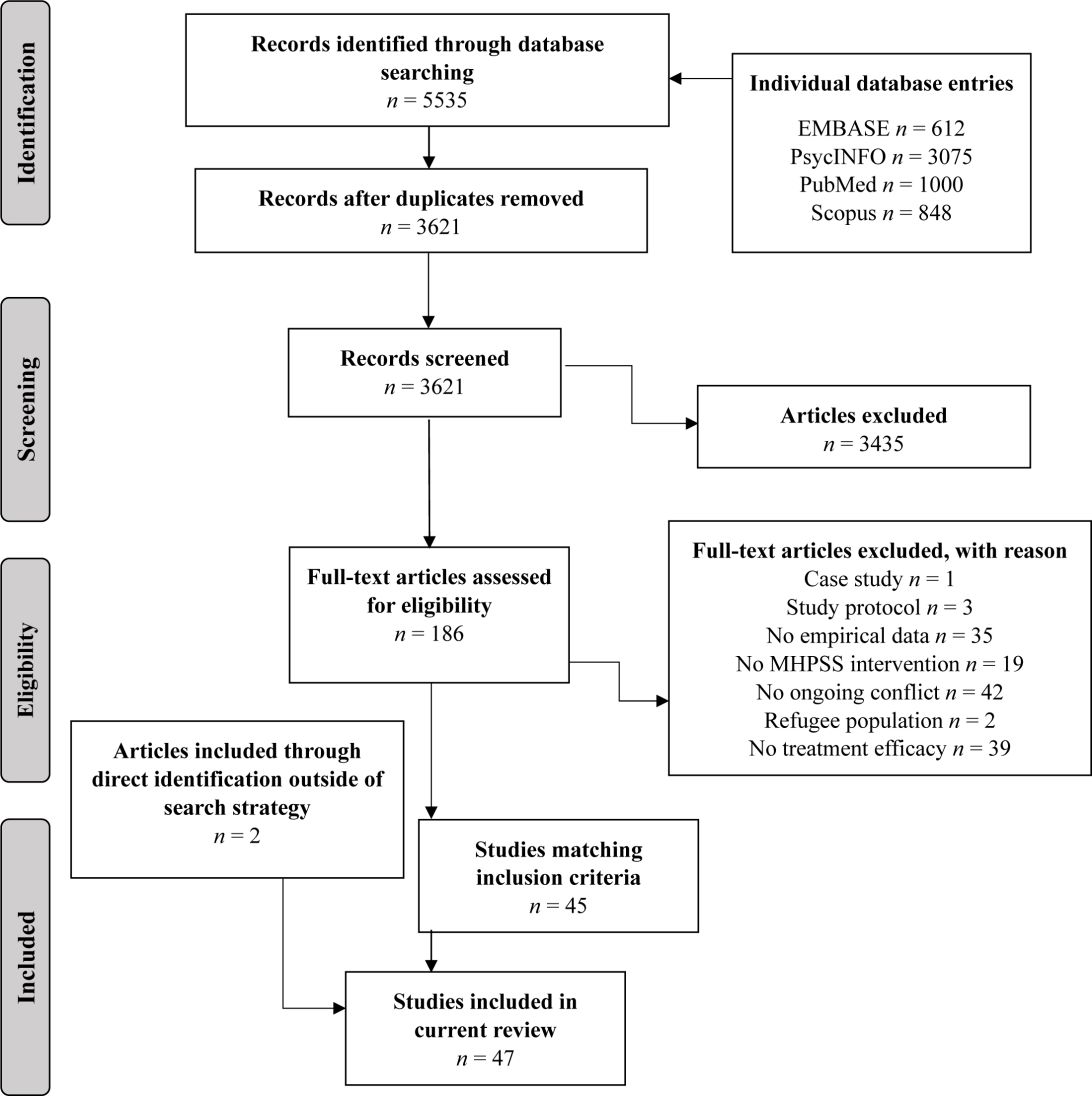
Flow diagram of scoping review. Edited from Moher *et al*.^130^ MHPSS, mental health and psychosocial support.

A total of 5535 papers were identified in EMBASE (n=612), PsycINFO (n=3075), PubMed (n=1000) and Scopus (n=848). After removal of duplicates, 3621 individual records remained. During the first round of selection based on titles and abstracts, 3435 papers were excluded and 186 remained for the second round of selection. After assessing the full texts for inclusion and exclusion criteria, 45 papers were judged suitable for this scoping review. An additional two papers were identified outside the search strategy and included in this review,^56 57^ making the total of included papers 47. Figure 1 depicts the selection process.

### Geographic coverage of studies

**Figure 2:**
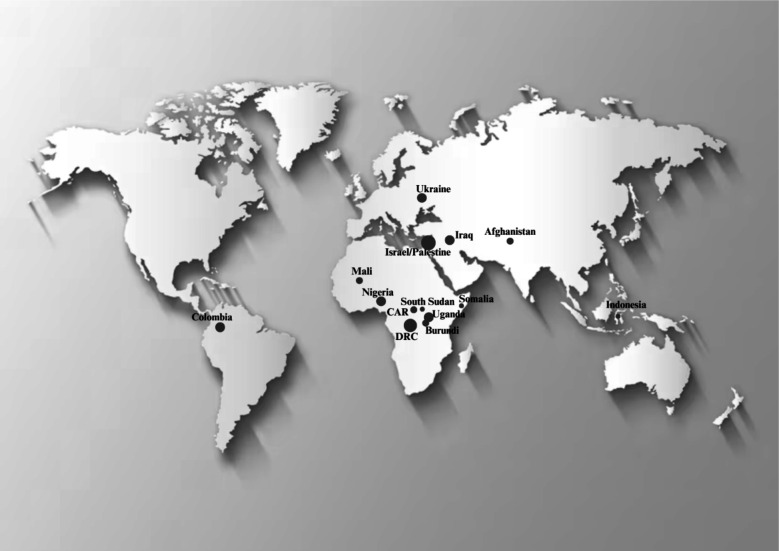
Overview of countries. Geographic distribution: Israel and Palestinian Territories n=16, DRC n=7, Colombia n=6, Iraq n=4, Ukraine n=4, Nigeria n=3, Uganda n=3, Mali n=2, South Sudan n=2, Burundi n=2, Afghanistan n=2, CAR n=1, Indonesia n=1, Somalia n=1. CAR, Central African Republic; DRC, Democratic Republic of the Congo.

Of the 47 included papers, most described studies conducted in the Middle East (n=22^58^,^79^), followed by Africa (n=14^80^,^93^), South America (n=6^94^,^99^), Europe (n=4^56 57 100 101^) and Asia (n=1^102^) (see figure 2 for a map of the geographic distribution). Given that some papers described more than one country, the overall count of countries is larger than the number of studies.

### Study populations

Of the included studies, 33 investigated MHPSS interventions targeting children and/or adolescents (under 18 years of age), of which 19 exclusively focused on children and adolescents (online supplemental table 1) and 14 included both children and/or adolescent and adult populations (online supplemental table 2). Of these, seven focused on IDPs. An additional 14 studies exclusively focused on exploring MHPSS interventions in adults, of which five focused on veterans and/or ex-combatants (online supplemental table 3).

### Child and adolescent interventions

19 studies investigated MHPSS interventions in a population that consisted exclusively of children and adolescents (online supplemental table 1). It is important to note that not all studies provided independent datasets. Six studies were conducted by the same research group, all focusing on school children living in the Gaza Strip,^6566 69 71^,^73^ and collectively reporting on only two distinct populations and interventions, with just one paper presenting unique data.^71^ Additionally, three other research groups also published two papers each, investigating either the same or an improved version of an intervention. In the case of Berger *et al*, their second paper^62^ investigated an extended version of the intervention tested in their first paper^61^ namely the ‘Enhancing Resiliency Amongst Students Experiencing Stress’ method (16 weeks) versus the ‘Overshadowing the Threat of Terrorism’ method (8 weeks).

#### Conflict: region, timeframe and context

The studies focusing on children/adolescents were predominantly implemented in the Middle East (namely, the Palestinian Territories (n=10), Israel (n=3) and Afghanistan (n=1)) and Africa (n=4, including Burundi, the Democratic Republic of Congo (DRC), the Republic of South Sudan and Uganda). Notably, the African studies were all carried out during periods of ongoing civil war or armed conflict with rebel groups—though after the peaks in armed conflict—and the study by Tol *et al*^102^ in Poso, Indonesia, was conducted in the context of ongoing sectarian violence between religious groups. In contrast, the studies in the Middle East were all completed shortly after periods of intensified violence, such as direct military assault or periods of major terror attacks. The intensity in terms of deaths and duration of armed conflict varied greatly between the studies: for example, all four African conflicts are considered especially deadly, with the Second Congo War and its aftermath estimated to have caused 5.4 million excess deaths between August 1998 and April 2007,^103^ and the wars in South Sudan, Burundi and Northern Uganda resulting in approximately 100 000 to 300 000 deaths each.^104^ In contrast, the Israeli-Palestinian conflict has historically been marked by episodic escalations, with death tolls ranging from dozens to over 2000 per episode, depending on the intensity and duration of the violence, with a sharp peak reaching over 20 000 fatalities since 2022.^105^ The conflict in Poso, Indonesia, while shorter in duration, resulted in an estimated 1000 deaths between 1998 and 2001 during periods of intense sectarian violence.^106^ None of the studies described specific measures taken by their staff to ensure safety during the research period, though Tol *et al*^93^ noted that all communes in areas populated by rebel forces were excluded from participation in the study.

#### Study population, intervention type and design

Of the 19 studies focusing on children/adolescents, 16 investigated a school-based group intervention; the 3 remaining studies investigated group interventions outside school settings.^64 76 83^ Sample sizes varied considerably from relatively small trials such as Ahmadi *et al*^58^ (n=63) to larger studies such as Shoshani^74^ (n=2228). Most studies focused on a civilian population, with two studies focusing on a population consisting of IDPs.^76 83^ The participants ranged in age from 7 to 18 years, with most studies focusing on middle childhood to early adolescence (age typically 10–13 years). Gender distribution was generally balanced, though most studies included slightly more male than female participants, and one study included female participants only.^58^

Only two of the studies on children investigated the efficacy of trauma-focused interventions, namely trauma-focused cognitive-behavioural therapy (CBT).^58 89^ For the 17 non-trauma-focused interventions, some did include specific sessions where the focus was more shifted towards trauma (eg, ^93 102^) and interventions integrated CBT-based trauma narrative restructuring into part of their sessions. Most studies adhered to a quasi-randomised trial or cluster design, and all except two^64 84^ included a control group to enable comparative estimation of the efficacy of the tested intervention.

#### Intervention providers and supervision

Most interventions, including the trauma-focused therapies, were implemented by trained non-mental health providers, including teachers, school counsellors, lay community workers or peer mediators. In nearly all cases, facilitators received continued supervision throughout the duration of the intervention from either mental health professionals—including clinical psychologists or experienced therapists—or senior research staff.

#### Intervention outcomes

Regarding the study outcomes, 13 studies investigated post-traumatic stress symptoms,^5861 62 69 71^,^73 76 89 93 102^ 8 depressive symptoms,^69 71 72 74 76 83 93 102^ and 5 symptoms of anxiety.^61 74 83 93 102^ Other outcome measures included psychological distress, emotion regulation, interpersonal functioning, prosocial behaviour, academic functioning and attitudinal aspects such as hope and positivity. All three trauma-focused interventions showed significant reductions in post-intervention symptoms, though only Ahmadi *et al*^58^ and O’Callaghan *et al*^89^ investigated PTSD (Veronese and Barola^77^ investigated life satisfaction and affect). The 16 non-trauma-focused interventions demonstrated mixed results, with most studies (n=10) showing some significant effects on their outcome measures; 6 studies^65 66 69 71 76 93^ did not find any significant improvements and in one case^66^ found that the population deteriorated.

Details of each of the studies are provided in online supplemental table 1.

### Interventions for children, adolescents and adults

#### Conflict: region, timeframe and context

In addition to the child and adolescent-focused studies identified during the scoping review, 14 studies included both children/adolescents (18 years and younger) and adults (18 years or older) in their interventions and assessments (online supplemental table 2). Studies were concentrated in a few regions including central sub-Saharan, eastern and western Africa (n=6, including Burundi, Central African Republic, DRC, Mali, Nigeria and Uganda), the Middle East (n=4, including Afghanistan, the Gaza Strip, Israel and Iraq), South America (n=2, namely Colombia) and Europe (n=1, namely Ukraine), with conflict intensity (total deaths due to armed conflict) varying greatly between studies. Little information was reported regarding the conflict contexts, or participant and researcher safety precautions, except for Ayoughi *et al*,^60^ which clearly noted information regarding the impact of armed conflict on the study (eg, that for safety reasons only one site could be included and that it was difficult to safely send personnel to that site).

#### Study population, intervention type and design

Of 14 studies focusing on both children/adolescents and adults, 8 included civilians (predominantly females, except one study), 3 civilians and IDPs (one predominantly female, the other male) and 3 IDPs only (predominantly females). Overall, the studies included a very large age range (0–93 years), with the majority of studies investigating non-trauma-focused interventions (n=12; 85.7%) and 2 exploring a trauma-focused narrative exposure therapy (NET) (n=2; 14.3%).^85 91^ Of the non-trauma-focused interventions, most consisted of unstructured or unspecified individual, family and/or community/group psychotherapies delivered in varying settings including Médecins Sans Frontières (MSF) or the International Committee of the Red Cross (ICRC) clinics, primary healthcare settings or IDP camps. Additionally, one intervention represented a culturally adapted form of CBT^92^ carried out with IDP populations.

With the exception of three studies,^60 85 92^ all studies were uncontrolled, of which three did not report study design.^68 91 98^ Of the controlled studies, one was a RCT,^85^ another was a pilot RCT^92^ and the third used pre-intervention/post-intervention comparison groups.^96^

#### Intervention providers and supervision

Most treatments were implemented by licensed psychologists and/or psychiatrists (n=3), lay counsellors (not licensed) with supervision from licensed psychologists or psychiatrists (n=6), and mixed teams of lay counsellors and other healthcare professionals such as licensed psychologists, psychiatrists, social and family workers (n=4). Only one study consisted of a small sample (n=16), while all others included samples between 75 and 14 963 participants.

#### Intervention outcomes

Studies investigated post-traumatic stress symptoms (n=8^59 68 81 82 85 91 96 101^ and complex PTSD n=1^68^), depressive symptoms (n=7^60 81 82 85 91 92 96^ and suicidality n*=*1^52^), anxiety symptoms (n=7^60 68 75 81 82 92 96^), alcohol use (n=1^96^), psychological distress and/or general mental health (n=4^63 68 88 98^) and psychosocial and/or daily life functioning (n*=*3^63 81 82^). Other study outcomes included externalising and internalising behaviours in children (n=1^63^), prosocial behaviours in adults and children (n=2^63 92^), parental self-efficacy (n=1^63^) and child/adolescent and parental coping and resilience (n=2^63 96^). Except for three studies,^63 96 101^ all others did not separately report outcomes for children/adolescents versus adults, and one article did not include the results from the adult population.^101^ For both trauma-focused and non-trauma-focused intervention studies, the majority reported medium to large effects of intervention on PTSD symptoms and secondary outcomes such as anxiety and depression.

Details of each of the studies are provided in online supplemental table 2.

### Adult interventions

#### Conflict: region, timeframe and context

14 studies investigated interventions in a population that exclusively included adults (18 years and older) (online supplemental table 3). The studies were concentrated in five geographic locations: Colombia (n=4), DRC (n=3), Iraq (n=3), Ukraine (n=3) and Somalia (n=1). Although some studies were carried out amidst armed conflict (eg, ^56 57 70 80^), the peak of violence usually preceded the research and some studies were carried out long after the conflict (eg, ^75 79 86 87 90 94 95 97 99^). Note that one study was carried out *before* the peak in armed conflict.^100^ Even for studies conducted around the peak in armed conflict, very little was reported about the exact condition that study participants (and healthcare providers) were exposed to at the time of treatment and the difficulties researchers experienced in conducting research during armed conflict.

The intensity in terms of deaths and duration of armed conflict varied greatly between studies, with, for example, Iraq representing a conflict with many deaths in a short period (151 000–601 000 estimated violent deaths between March 2003 and June 2006^107 108^) and Colombia representing a conflict with fewer deaths over a long period (42 000 estimated violent deaths between 1989 and 2023^109^).

#### Study population, intervention type and design

Of the 14 studies focusing on adults, 9 studies included civilians (predominantly female), 5 studies included veterans/ex-combatants (predominantly male) and no studies included IDPs. The sample size varied considerably between studies (range 15–3740) and five studies included a sample with less than 50 participants in total. Just over half of the studies on civilians investigated a trauma-focused intervention,^7078^,^80 90 94^ usually a form of trauma-focused cognitive behavioural interventions or NET (n=6; 66%), while the remaining studies (n=3; 33%) investigated a non-trauma-focused intervention.^57 95 97^

Two of the studies focusing on veterans/ex-combatants investigated a trauma-focused intervention in the DRC (adapted versions of NET; FORNET) focused on PTSD symptoms,^86 87^ and the remaining three investigated non-trauma-focused interventions aimed at general stress reduction, psychopathology and emotional processing in Colombia and Ukraine.^56 99 100^ All veteran studies were controlled, usually using a treatment as usual comparison condition (although one study did not include randomisation, and one study used pseudo-randomisation and reported positive findings with large effect sizes).

#### Intervention providers and supervision

Most interventions were implemented by trained local lay counsellors, except for one study where treatment was provided by local licensed psychologists. More specifically, trauma-focused interventions were provided by locally trained and supervised psychologists/psychiatrists or lay community workers, while non-trauma-focused interventions were almost exclusively provided by locally trained and supervised community workers/counsellors or students. One study did not specify the nature of providers^56^ and one study used a digital chatbot provider.^57^

#### Intervention outcomes

Common outcomes included PTSD (n=10^7078^,^80 86 87 94 95 97 100^), depression (n=9^5670 78^,^80 86 94 97 100^), anxiety (n=8^56 70 78 79 94 95 97 100^), quality of life and well-being (n=5^7078^,^80 97^) and psychosocial functioning (n=1^99^). Other outcomes included general mental health (n=2^94 97^), perceived stress (n=2^57 79^), somatic symptoms (n=2^80 95^), victimisation and perpetration (n=1^90^) and aggression and violence (n=3^86 87 99^). In veterans or ex-combatants, the following additional outcomes were also investigated: drug dependence (n=1^86^) and emotional processing and social skills (n=1^99^). All studies on trauma-focused interventions (n=8, of which 2 did not include any control condition) reported large significant effects of the intervention on PTSD symptoms and many reported significant effects on secondary outcomes such as depression and anxiety. Similarly, all studies on non-trauma-focused interventions (n=6, of which 1 was uncontrolled) reported large significant effects of the interventions on functioning and distress.

Details of each of the studies are provided in online supplemental table 3.

## Discussion

This scoping review sought to capture the current quantitative evidence base pertaining to MHPSS interventions for populations affected by ongoing armed conflict and war since the post-Cold War period.

### Intervention findings: scope—what, who, where, how?

Findings demonstrate a wide variation in the types of interventions, outcome targets, assessment instruments and delivery settings covered by the extant literature. MHPSS interventions can be grouped into four broad categories: in *adults*, (1) trauma-focused CBT or NET interventions aimed at reducing PTSD symptoms in civilians and ex-combatants as well as aggression in the latter group, (2) non-trauma-focused psychotherapies and psychosocial support interventions to improve functioning and reduce overall distress; in *children and adolescents*, trauma-focused and non-trauma-focused (3) group, school-based psychosocial interventions and (4) adolescent group therapy focused on diminishing PTSD symptomatology and interpersonal aggression, and improving overall well-being, family and peer relations, and prosocial behaviours.

Two-thirds (67%) of the included studies described children and adolescent interventions, of which 41% included both children/adolescent and adult populations. The remaining one-third (33%) of all included studies focused on adults, with nearly half assessing an evidence-based treatment (EBT) in line with American Psychological Association (APA),^110^ National Institute for Health and Care Excellence (NICE)^111^ and International Society for Traumatic Stress Studies (ISTSS)^112^ guidelines. Only two articles focusing exclusively on children/adolescents investigated an EBT—namely trauma-focused CBT^58 89^—while nearly one-third of studies including both children/adolescent and adult populations assessed EBTs. The remainder examined psychosocial activities and unspecified individual, family and/or group psychotherapies aimed at reducing PTSD and other trauma-related symptoms and improving functioning, coping and resilience, including among children-parent dyads.^63 96^

While a larger proportion of studies focused on children/adolescents, many were conducted by the same research groups using identical samples. Overall, few studies included infants, young children, older adults aged 65 years or over, or vulnerable populations such as LGBTQI+ and people living with disabilities or with neurological disorders. Though female participants accounted for a larger proportion of study samples, two exclusively focused on females^58 79^ and a few included male ex-combatants only (eg, ^56 86 87^). No studies included gender breakdowns or focused on gender-expansive populations.

Despite the challenging conditions under which the included studies were undertaken, nearly two-thirds (61%) included large sample sizes (**≥**200), pointing to the feasibility of participant recruitment into large-scale studies (eg, RCTs) under conditions of ongoing armed conflict. Several studies were conducted by, or in partnership with, international non-governmental organizations (NGOs) with local in-country presence, highlighting the importance of meaningful partnerships between researchers and not-for-profit organisations active on the ground. Only four studies leveraged the use of technology (chatbot, virtual reality, digital clips and songs, CBT delivered via phone) for intervention delivery.^56 57 74 75^

Most studies were undertaken in non-mental healthcare settings such as primary healthcare clinics, schools, places of worship or community gathering. Similarly, the majority of interventions were delivered by non-mental healthcare professionals (53%), providing an important foundation both for the acceptability and scalability of MHPSS interventions. The number of intervention sessions ranged from 1 to 19 sessions, with session frequency varying from weekly to several months. Common MHPSS intervention components included psychoeducation, stress management and activities aimed at diminishing distress and enhancing psychosocial functioning, quality of life and/or community reconciliation through coping, resilience and prosocial skills building. Other common MHPSS activities included psychological first aid, individual, family and group psychotherapy, and/or cognitive and emotional processing techniques to reduce PTSD and trauma-related symptoms such as depression and anxiety.

Geographically speaking, few studies focused on Western Africa, Southeast Asia or Latin America (other than Colombia). In fact, a geographic ‘clustering’ appears with a disproportionate number of interventions implemented in Colombia, DRC and the Palestinian Territories (specifically, the Gaza Strip), at times reported by the same research group(s). The included studies were conducted in 15 different countries, most of which (53%) had conflicts persisting for over 30 years; 47% of the countries had conflicts enduring between 10 and 30 years, and none lasting 10 years or less. While the rationale for site selection was not explicitly outlined in the included articles, geography-specific intervention density may reflect broader geopolitical dynamics affecting research financing and overseas development aid flows at the time of research and/or advocacy by proactive local or diaspora groups with global visibility. Other potential factors may include on-the-ground security and safety considerations, community acceptance affecting data collection feasibility, and the existence of prominent research groups with strong country- or community-specific ties.

Most studies were carried out in the community with civilian populations, with fewer conducted in refugee or IDP settlements and/or with ex-combatants. A larger number of studies were implemented in urban versus rural areas where access to infrastructure and care may be more prohibitive, though contextual details surrounding intervention settings and intersectoral integration were frequently lacking.

#### Cultural relevance of interventions

While most interventions were delivered by lay local trainers in local languages, many were originally developed in Western, Educated, Industrial, Rich, and Democratic (WEIRD) countries (eg, CBT, NET) and transposed onto local settings with minimal documented evidence of community consultations and contextual or cultural adaptation. Furthermore, local trainers were typically supervised by qualified psychologists or psychiatrists originating from WEIRD settings, with little information concerning trauma- and context-informed approaches. Similarly, assessment tools were frequently translated without describing specific attunement to cultural idioms of trauma, distress or healing. In addition, despite some exceptions (eg, ^59 80^), the majority of studies included outcomes conceptualised using a western, biomedical framework. Few assessed culturally specific trauma sequelae such as somatic or psychosomatic symptoms at individual levels, community fragmentation or community-level cultural resource loss, and broader societal implications such as erosion of social fabric, inter-communal conflicts, mistrust and embitterment.

These omissions should not necessarily be interpreted as an absence of cultural adaptation but rather point towards ongoing gaps in systematic reporting of these processes in the existing literature. Resource and research timeline constraints under strenuous conditions of ongoing conflict may also account for some of these gaps in cultural adaptation. One interesting example of cultural adaptation reported in the included studies involves the use of a participatory research tool—the Wellbeing Exercise in War Child Holland’s I DEAL psychosocial intervention.^84^ Through a reflection exercise surrounding the meaning of child well-being in the community and the behaviours associated with peers who are ‘doing well’, this tool allowed researchers to compare intervention content with local South Sudanese children’s perceptions of well-being and trauma. Another example is the study by O’Callaghan and colleagues^89^ which engaged a community-based committee trained in child protection and psychosocial support (Reseaux Communautaires pour la Protection de l’Enfance) to provide ethical and cultural advice on the study and to administer the questionnaires. Despite these examples, however, few studies explicitly included qualitative information pertaining to study setting and conflict status or needs assessments and community engagement processes. The inclusion of more systematic cultural and contextual adaptation processes and their impact on intervention effectiveness is a point that would benefit from additional research.

#### Intervention effectiveness

Many studies reported statistically significant positive outcomes in both adult civilians and ex-combatants such as decreased symptomatology of trauma-related disorders including PTSD, depression and anxiety symptoms as well as broader psychosocial measures of overall functioning, well-being and quality of life. Findings were however mixed when considering reductions in aggression, drug dependence and improvements in social/interpersonal skills among adult ex-combatants. These results may highlight potentially differential mechanisms at play among internalising versus externalising disorders as well as gendered effects (eg, for exclusively male vs predominantly female samples), which interact with both index trauma and trauma recurrence to predict treatment response and effectiveness. Contextual elements may also explain finding variations, including the complexity of demobilisation, disarmament and reintegration (DDR) efforts within the context of conflict resolution and peacebuilding efforts. During active armed conflict, DDR efforts may be nascent or non-existent, resulting in MHPSS interventions being provided in silo or too early in the conflict cycle when post-conflict recovery has not yet occurred or the window of reconciliation is too close. Additional contextual considerations may include intervention provider sex, gender or background including ethnicity, religion, and whether they are a member of the victim/perpetrator community or a recovered ex-combatant.

While the impact of cumulative exposure to potentially traumatic events (PTEs) was often not explicitly measured in the included articles, it should be considered given its association with greater PTSD risk and symptom severity^113^ and lower probability of remission.^114^ Recurrent exposure to PTEs has been shown to interact with the index trauma, with recurrence of the same trauma type in *victims* of (physical) violence, associated with increased PTSD risk.^115^ Conversely, recurrent exposure to PTEs involving *perpetration* of violence—including combat, perpetration of injury or death—has been linked to decreased PTSD vulnerability.^115^ This is consistent with first, low PTSD prevalence findings among combatants demonstrating appetitive aggression^116^ and second, improved performance in the police force^117^ and first responders^118^ during high stress conditions, supporting the notion of potentially differing neurobiological PTSD pathways dependent on the index trauma.^119 120^ Additionally, the role of potentially morally injurious events can also not be excluded where events associated with ‘others’ responsibility’—such as in the case of sectarian violence—tend to be associated with externally directed cognitions and emotions such as anger and lack of other-forgiveness.^121^ In this review, no included studies investigated moral injury or complex PTSD (CPTSD) as either a primary or secondary outcome, possibly due to the novelty of the latter’s diagnostic category but nonetheless a worthwhile avenue for future research across diverse populations.^122^ Similarly, a strong focus remained on post-traumatic conditions and symptoms despite occurring in peri-traumatic contexts of ongoing armed conflict.

In children and adolescent civilian populations, intervention study findings on trauma-related disorder symptomatology, including PTSD, depression and anxiety, as well as those examining prosocial and aggressive behaviours were also mixed. Study effects often failed to reach statistical significance or showed negative effects, including a worsening of interpersonal conflict or aggression, lack of reduction in conduct problems, decreased prosocial behaviours and increased PTSD symptoms. One possible explanation may be that none of the child/adolescent interventions included EBTs as defined in APA, NICE and ISTSS guidelines.^110^,^112^ Another explanation may be that assessment tools developed in WEIRD contexts fail to capture culturally specific trauma syndromes and idioms of distress (see, for example, ^123 124^) and to account for the role of cultural factors in PTSD prevalence (see, for example, ^125^). Intervention delivery settings and modalities may also have influenced variations in observed outcomes, for example, resulting from implementation inside versus outside the classroom, in overcrowded homes or schools versus child friendly spaces, or by a teacher versus trained mental healthcare professional of the same versus different sex. This highlights the need for both a better understanding of trauma phenomenology across cultures and for culturally and contextually validated, transdiagnostic measures—including of clinical worsening. Cultural and contextual adaptation grounded in local, community needs is particularly important to safeguarding against the risk of causing undue harm through MHPSS intervention implementation^126^, including preventing the risk of pathologising trauma, unintentionally causing distress and perpetuating cycles of violence, in line with the IASC recommendation pertaining to the ‘do no harm’ principle.^24^

#### Research and methodological standards

Overall, methodological standards were relatively low making it difficult to draw strong conclusions regarding intervention effectiveness. Methodological concerns include dropout rates—at times high and rarely justified, few active control conditions included in RCTs, and few uses of (culturally) validated assessment instruments employed to measure changes in both psychological and psychosocial outcomes. Moreover, while several studies included a waitlist condition for which outcomes were reported, others measured overall intervention effectiveness through binary yes/no responses from participants or trainers, contributing to overall low-quality evidence. In the case of MHPSS interventions targeting children/adolescents, not all studies provided independent datasets, with multiple studies conducted by the same research group(s).

In addition, qualitative information rarely complemented quantitative data, at times making it difficult to assess inclusion criteria and limiting the replicability of studied interventions. For example, information surrounding population and site selection, community access and engagement mechanisms, cultural adaptation processes, intervention components, descriptions of delivery settings and procedures to ensure participant and researcher safety were rarely reported. Definitions of *armed conflict* were scarce, varying and at times interchanged with *war* terminology. Few studies provided details surrounding conflict settings, timeframes and intensity, including the level of violence at the time of implementation and how these overlapped and affected study periods (eg, periods of ceasefire vs active conflict). As a result, the authors of this review needed to infer, and research, additional contextual details concerning the status of studied conflicts. While these gaps do not necessarily imply a lack of intervention efficacy, they do point to the need for additional, rigorous scientific research standards and evidence-based quality assurance mechanisms to facilitate comparability across populations and settings.

#### Limitations

Several limitations must be noted when interpreting current findings. First, the review neither includes empirical studies conducted prior to 1991 nor published in a language other than English; both restrictions were selected to limit the scope of the present paper. Second, grey literature was not included as part of the scoping review and thus did not capture MHPSS activity and intervention reports published outside of peer-reviewed, academic journals. Third, while most interventions address PTSD symptoms and/or psychological distress resulting from exposure to conflict-related traumatic events, diverse intervention types conducted under varying conflict dynamics, duration and intensity and influenced by heterogeneous sociocultural and politico-historical contexts were included. Similarly, the review did not control for the type of traumatic event, symptom duration or severity, nor the length of time since trauma exposure, all of which may affect outcomes.

#### Recommendations

Based on the findings of the current review, we outline several recommendations to help address identified evidence gaps and strengthen research, monitoring and evaluation systems for MHPSS in active armed conflict settings. First, specific attention should be given to the conceptual paradigms used when assessing needs and designing interventions, ensuring that these are grounded in an understanding of local populations, contextual nuances and culture. While a discussion of existing MHPSS paradigms is beyond the scope of this review, frameworks seeking to safeguard participants’ inner resources and autonomy and empower communities to (re-)build their own psychological, social and cultural resources to heal may go a long way in reducing unintended harm, promoting recovery and fostering pathways to peace in conflict settings (see for a broader debate, for example, ^30 31^). The dissemination and application of findings *following* the research process are also important both to enhance participant retention for longitudinal follow-up and to ensure intervention design and evaluation are grounded in, and informed by, local needs.

Second, the implementation of MHPSS interventions in non-specialised mental healthcare settings (eg, community centres, places of worship, schools), including the integration of EBT components into existing, non-health-specific activities (eg, education, spiritual practice) represents an important entry point to addressing stigma surrounding seeking and accessing treatment. Such intersectoral approaches may also open new avenues for local ownership and sustainable MHPSS system strengthening when codeveloped through meaningful engagement of conflict-affected communities, grounded in reciprocal learning and implemented through a phased approach.^127^ Similarly, task shifting within health and social workforce teams, and capacity building of lay community members supported by digital tools, represents an important means of overcoming challenges related to gaps in trained mental health human resources and mental health system financing. Together these approaches may help lay the foundation for effective post-conflict mental health and psychosocial service integration into broader health, education, social protection and other national systems, while presenting a more resilient, cost-effective pathway to scaling as conflict lines evolve.

Third, while conflict setting and population idiosyncrasies will, and should, continue to guide MHPSS intervention development and implementation (see e.g. Mc Mahon et al^126^ for practical strategies), more research is needed surrounding the delicate balance of *psychosocial* and *mental health* intervention components—including best practices in psychoeducation which are currently largely lacking and how these impact broader conflict resolution dynamics. A better understanding of how these intervention components interact—including their mechanistic underpinnings, to affect mental health outcomes at individual, interpersonal and community levels on the one hand, and collective healing, well-being, social identity and reconciliation at a societal level on the other hand—is needed. From a policy perspective, linking micro-level and macro-level outcomes by placing these within broader conflict resolution, transitional justice and peacebuilding frameworks provides a meaningful contribution to understanding the intricate interdependencies between mental health, cycles of violence and conflict perpetuation and may offer new possibilities for sustainable pathways to enduring peace.

Fourth, an understanding of the ‘key ingredients’—or core therapeutic components—underpinning an integrated approach to EBTs and psychosocial interventions such as community reconciliation and conflict resolution programmes in contexts of ongoing conflict is needed to inform scaling and replication both within and across contexts and population groups. Systematic approaches to embedding or adapting EBTs within a broader multilevel framework accounting for the unique impact of structural factors, politico-historical processes and systemic power dynamics on individuals, their families, communities and societies are needed. Such frameworks may be particularly useful when conceptualizing these ‘key ingredients’, and attuning them to, unique and changing conflict-affected contexts as a means of identifying relevant intervention entry points and reducing unintended harm.

Fifth, the validation of both standardised and culturally adapted clinical and psychosocial assessment tools and measures—with a focus on transdiagnostic instruments, across contexts, populations and cultures—is needed to both capture the broad array of trauma-related symptoms and psychosocial impairments and assess clinical worsening among conflict-affected populations. Assessment tools and EBT approaches aimed at addressing cumulative, and often complex, conflict-related traumatic experiences are required to both improve mental health and psychosocial outcomes and address recurring cycles of violence. In this respect, a better understanding of the differential mechanistic pathways underpinning vulnerability to varying trauma-related disorders versus other externalising behaviours such as appetitive aggression or revenge-seeking—including their relationship with specific types of PTEs, potentially morally injurious events and potential gender differences—will be crucial to informing preventive interventions and disentangling mutually reinforcing cycles of violence and mental ill-health.

Sixth, additional methodological rigour is required. The wide heterogeneity of research designs, results and clinical instruments used to assess changes in mental health and psychosocial outcomes across the included studies points to the need for improved research standards when evaluating MHPSS interventions in ongoing conflict settings. This may include a focus on longitudinal studies designed to assess changes in PTSD symptoms, psychosocial outcomes and/or culturally specific trauma sequelae over time as well as the prospective impact of early interventions on community reconciliation, the reduction of social divisiveness and the prevention of trauma-related disorders.^6^ The integration of moderation and mediation assessment studies to determine how study outcomes are (or are not) reached may also be helpful when gold standard RCTs are not feasible.^128^

#### Future research directions

One potentially fruitful avenue to strengthening MHPSS research standards may be to develop a commonly agreed upon minimum set of quality standards which balance methodological rigour with contextual feasibility, sanctioned by the research community and on-the-ground practitioners alike. Such standards would help elevate the minimum quality of MHPSS research while accounting for both resource and research constraints. In this way, ‘good enough’ research standards could be achieved, bridging research and practice, and facilitating outcome replication and comparability across conflict settings. Such standards may include the identification of a brief, common set of assessment instruments for use across studies to facilitate outcome comparison and intervention effectiveness measurement. These may also support the creation and application of a common instrument development process and cultural adaptation protocol across sociocultural contexts and conflict settings.^6^ A more detailed account of such standards can be found in the forthcoming position paper informed by this scoping review.^129^

## Conclusion

The current review summarises existing evidence on MHPSS interventions for populations affected by ongoing armed conflict and war since the post-Cold War period, identifies evidence gaps and proposes strategies to strengthen research, monitoring and evaluation in these settings. Many studies reported statistically significant positive outcomes in both adult civilians and ex-combatants including decreased PTSD, depression and anxiety symptomatology and improved psychosocial functioning, well-being and quality of life. Results were more mixed in children/adolescents and when examining aggression, drug dependence and social/interpersonal skills in ex-combatants, highlighting the need for additional research.

Most studies included large sample sizes and control conditions despite potential challenges associated with accessing populations and ensuring participant and researcher safety amidst active armed conflict. This review also revealed gaps in the systematic reporting of study design, implementation context, nature and timeframe of the conflict, cultural adaptation, community engagement and acceptance. The wide heterogeneity of interventions, assessment scales, outcome targets and delivery settings also presents significant challenges for comparability, replicability and scale. A minimum set of quality research standards may support the creation of shared research frameworks allowing for common instrument development and cultural adaptation protocols that are replicable and comparable across settings. Over time, these standards may facilitate the identification of shared, replicable intervention elements for scale.

In conclusion, this review reveals that MHPSS intervention research, while challenging, is feasible in contexts of active armed conflict. A better understanding of interventions effectiveness in settings of ongoing conflict is crucial to advancing the MHPSS field and ensuring it is systematically grounded in a robust evidence base. Only in this way can we maximise increasingly finite resources to reduce psychological distress, promote mental health and psychosocial well-being and prevent undue harm for populations worldwide.

## Supplementary material

10.1136/bmjgh-2025-022708 online supplemental file 1

10.1136/bmjgh-2025-022708 online supplemental file 2

## Data Availability

Data sharing not applicable as no datasets generated and/or analysed for this study.
