# Supplementary material for: Mental health and psychosocial support interventions for populations affected by ongoing armed conflict: a scoping review
Source: BMJ Glob Health. 2026 May 27;11(5):e022708. doi: 10.1136/bmjgh-2025-022708 (PMC13218107; doi:10.1136/bmjgh-2025-022708)
Supplement: online supplemental file 1 [file bmjgh-11-5-s001.docx]

**Supplementary Table 1. MHPSS interventions targeting children and/or adolescents**

| **Study** | **Location** | **Design** | **Participants** | **Intervention and control** | **Provider and supervision** | **Targets** | **Outcomes** |
| --- | --- | --- | --- | --- | --- | --- | --- |
| **Civilians - Trauma-focused school-based interventions** | | | | | | | |
| Ahmadi et al. (2024) [1] | Afghanistan | Quasi-experimental research design | *N* = 63 (100% female), *M*_age_ = 14.80, age range 12-18  *N*_TF-CBT_ = 20,  *N*_ACT_ = 20,  *N*_waitinglist_ = 23 | 1. TF-CBT for 7 sessions of 75 minutes each, held three times a week (every other day) for two weeks, and a last meeting in the third week.  2. ACT for 8 sessions of 75 minutes each, held every other day for two weeks and twice in the third week. 3. Waiting list | Trained facilitator under supervision of first author (a psychologist) | Posttraumatic stress symptoms (CRIES-13) | The difference in mean PTSD score between baseline and post-test was 18.3 for the ACT group and 11.8 for the TF-CBT group (Cohen’s d respectively 3.07 and 2.55). The difference between ACT and TF-CBT was not statistically significant (p = 0.52) |
| O’Callaghan et al. (2015) [2] | Democratic Republic of Congo | Equal randomization, single blind, parallel group intervention | *N* = 72 (42.0% female), *M_age_* = 14.88, age range 14-17  *N*_TF-CBT_ = 26,  *N*_CFS_ = 24 | 1. TF-CBT for 9 sessions of 1.5 hours, three times per week 2. Child Friendly Spaces for 9 sessions of 1.5 hours, three times per week | Non-clinically trained, indigenous facilitators, supervised by on site lead researcher (a psychologist). | PTSD (UCLA PTSD- Index), internalising symptoms, conduct problems and prosocial behavior (African Youth Psychosocial Assessment Instrument) | Using pre-intervention PTSD scores as covariant, there was a significant reduction in post-intervention PTS symptoms (F(2,68)=40.40; P<0.001), but no difference between interventions.  Similarly, internalising symptoms and conduct scores were significantly reduced post-intervention, but not different between interventions.  Using ANCOVA with pro social behaviour as covariant, there were no significant reductions in conduct problems between the groups at post-test. However, at 6 months follow-up the Child Friendly Spaces condition had a significant reduction in pro social behaviour compared to the TF-CBT group. |
| **Civilians - Non-trauma-focused school-based interventions** | | | | | | | |
| Berger et al. (2007) [3] | Israel | Quasi-randomized controlled trial | *N* = 154 (53.9% female), *M_age_* = 12.8, age range 11-13  *N*_intervention_ = 107  *N*_waitinglist_ = 47 | 1. Overshadowing the Threat of Terrorism class-room program of 8 weekly 90-minute sessions. 2. Waiting list | Trained local teachers, supervised by experienced therapists | PTSD (UCLA PTSD Index)), functional problems (items derived from the DISC), somatic complaints (DPS), general anxiety and separation anxiety (SCARED) | There were significant reductions on all measures of PTSD symptomatology, somatic complaints, and in both generalized and separation anxiety levels compared to the control group. |
| Berger et al. (2012) [4] | Israel | Quasi-randomized controlled trial | *N* = 154 (53.9% female), *M*_age_ = 12.8, age range 11-13  *N*_intervention_ = 107  *N*_waitinglist_ = 47 | 1. Extended Enhancing Resiliency Amongst Students Experiencing Stress class-room program of 16 weekly 90-minute sessions. 2. Waiting list | Trained local teachers, supervised by experienced therapists | PTSD (UCLA PTSD Index), functional problems (items derived from the DISC), somatic complaints (DPS), general anxiety and separation anxiety (SCARED) | The intervention group had a 2.7 higher odds of recovering from PTSD, whereas the control group had a 3.5 higher odds of experiencing a PTSD symptom elevation of more than 10%.  There was also a statistically significant improvement in the intervention group compared with the control group regarding separation anxiety (F(1,152) = 3.74, η2 = 0.15), functioning (F(1,152) = 19.05, η2 = 0.12), somaticism (F(1,152) = 18.30, η2 = 0.12), and general anxiety (F(1,152) = 22.8, η2 = 0.06). |
| Diab et al. (2014) [5] | Gaza Strip, Palestinian Territories | Randomized controlled trial | *N* = 482 (49.4% female), *M_age_* = 11.29, age range 10-13  *N*_intervention_ = 242  *N*_waitinglist_ = 240 | 1. Teaching Recovery Techniques program (extracurricular) of unknown duration and frequency. 2. Waiting list | Counselors with master's degree in psychology, supervised by clinical psychologist | Peer relations (CLS and FQS), sibling relations (Dunn’s questionnaire for sibling interactions), posttraumatic symptoms (IES-R), depressive symptoms (DSRSC), psychological distress (SDQ), psychosocial well-being (MHC-SF) | The intervention was not associated with statistically significant increase in the level of wellbeing or prosocial behaviour among children who reported exposure to two or more types of trauma. Instead, the intervention was statistically significantly associated with a decrease in the proportion of children above the cutoff score of prosocial behaviour. |
| Diab et al. (2015) [6] | Gaza Strip, Palestinian Territories | Randomized controlled trial | *N* = 482 (49.4% female), *M_age_* = 11.29, age range 10-13  *N*_intervention_ = 242  *N*_waitinglist_ = 240 | 1. Teaching Recovery Techniques program (extracurricular) of unknown duration and frequency.  2. Waiting list | Counselors with master's degree in psychology, supervised by clinical psychologist | Psychotraumatic exposure (DSM-5 criterion A list), psychosocial wellbeing (MHC-SF)), prosocial behavior (SDQ), maternal attachment (Willingness to Serve as Secure Base for the Child), family atmosphere (Family Ambiance Scale) | Sibling conflict did not significantly decrease in the intervention group, but also did not increase. However, they did significantly increase in the control group, especially among boys (interaction effect of group x gender x change — F(2, 480) = 3.95, p < .05, η2 = .02).  Loneliness decreased in the intervention group only among boys, but not among girls. In turn, a decrease in sibling rivalry decreased in the intervention group only among girls.  There was a general deterioration in positive sibling relations, shown in decreased warmth and intimacy from baseline T1–T3 in both groups. Counter to our hypotheses, the intervention was not able to prevent that deterioration. |
| Eiling et al. (2014) [7] | Republic of South Sudan | Mixed-methods, non-randomized, pre-post exploratory evaluation | *N* = 122 (40.2% female), *M_age_* = 11.56, age range 8-16  *N*_intervention_ = 122  NB: no control group | 1. I DEAL life skills intervention of maximum 19 sessions of 1.5 hours over 4-6 months | Community workers; supervision not described | Local perceptions of wellbeing (Wellbeing Exercise), personal goals (Personal Goal Exercise), intervention satisfaction (CSQ-8) | During evaluation interviews, 48% of the respondents stated that they noticed significant personal improvement, while 30% noticed some improvement. Specifically, decreased fighting and improved relationships with peers and parents were reported. |
| Forsberg & Schultz (2023) [8] | Gaza Strip, Palestinian Territories | Randomized control trial | *N* = 300 (50% female), *M_age_* = 12.01, age range 9-17  *N*_intervention_ = 200  *N*_waitinglist_ = 100 | 1. Better Learning Program level 2 of 5 sessions of 45 minutes.  2. Waiting list | Trained school counselors with bachelor degree in psychology, supervised by Ministry of Education counsellors and clinical trainers with masters degree in psychology or education | Well-being, self-regulation, self-efficacy, executive function, future hope and self-perceived academic functioning (specifically developed self-report), stress-related symptoms (CRIES-13), academic functioning (grades in Arabic and math) | The intervention led to significantly higher well-being both at T2 and T3 compared to T1 (p < .00). It also led to significantly higher scores in self-regulation at T2 compared to T1 (p < .00), but not from T2 to T3 (p = 0.78). Lastly, the intervention led to significantly higher self-efficacy at T2 compared to T1.  There were no significant changes for the waiting list students. |
| Kangaslampi et al. (2016) [9] | Gaza Strip, Palestinian Territories | Cluster randomized trial | *N* = 482 (50.2% female), *M_age_* = 11.29, age range 10-13  *N*_intervention_ = 242  *N*_waitinglist_ = 240 | 1. Teaching Recovery Techniques program (extracurricular) for 8 sessions of 2 hours twice a week.  2. Waiting list | Trained counselors, supervised by senior research group member | Posttraumatic stress symptoms (CRIES-13), posttraumatic cognitions (cPTCI), depression (DSRSC), exposure to war trauma (specific checklist based on local situation and DSM-IV-TR criterion A) | Linear regression accounting for T1 parental trauma concerns (PTC) found no significant effect of the intervention at T3 or T4, and no gender differences.  Path analysis model explained 11% of the variance in post-traumatic stress symptoms (PTSS) at T4. PTC at T3 were significantly related to PTSS at T4 (β = 0.29) with no gender differences.  However, there was no significant effect of the intervention on PTSS at T4 through changes in PTC at T3. |
| Peltonen et al. (2012) [10] | Gaza Strip, Palestinian Territories | Cluster randomized trial | *N* = 225 (36.0% female), *M_age_* = 11.37, age range 10-14  *N*_intervention_ = 141  *N*_waitinglist_ = 84 | 1. School Mediation Interventions of unknown duration and frequency. 2. Waiting list | School mediators (students) with support from local teachers, supervised by the mental health team from the Gaza Community Mental Health Programme | Military trauma (specific checklist), PTSD symptoms (CPTS-RI), depression (CDI), psychological distress (SDQ), friendship quality (Bulkowski friendship questionnaire), prosocial behaviour (SDQ), and aggression (MAQ). | The intervention did not decrease PTSD, depression, or psychological distress. More importantly, the intervention group showed an increase in PTSD symptoms yet no change in the control group (while PTSD levels were comparable between groups). For depression and psychological distress, the control group exceeded clinically significant threshold at T2, whereas the intervention group did not.  The intervention was also not effective at increasing friendship quality, prosocial behaviour and nonaggressive behaviour. The control group showed deteriorated social functioning between T1 and T2. |
| Punamäki et al. (2014) [11] | Gaza Strip, Palestinian Territories | Randomized control trial | *N* = 482 (49.4% female), *M_age_* = 11.29, age range 10-13  *N*_intervention_ = 242  *N*_waitinglist_ = 240 | 1. Teaching Recovery Technique (extracurricular) for 8 sessions of 2 hours twice a week | Counselors with master's degree in psychology, supervised by clinical psychologist | Emotion regulation (ERQ), war trauma (specific event list), posttraumatic stress symptoms (CRIES-13), depressive symptoms (DSRSC), psychological distress (SDQ), psychosocial well-being (MHC-SF) | The intervention led to decreased emotion regulation from T1 to T3. This was associated with improved mental health (decreased symptoms and increased well-being). However, it was not effective in increasing control-enhancing emotion regulation strategies.  There was a decrease in intensity of fear, sadness, and anger in both the intervention and control groups. |
| Qouta et al. (2012) [12] | Gaza Strip, Palestinian Territories | Cluster randomized controlled trial | *N* = 482 (49.4% female), *M_age_* = 11.29, age range 10-13  *N*_intervention_ = 242  *N*_waitinglist_ = 240 | 1. Teaching Recovery Technique (extracurricular) for 8 sessions of 2 hours twice a week. 2. Waiting list | Counselors with master's degree in psychology, supervised by clinical psychologist | Peritraumatic dissociation (PDEQ), posttraumatic stress symptoms (CRIES-13), depressive symptoms (SDRSC), psychological distress (SDQ) | Low, medium, and high classes of peritraumatic dissociation had different parameter estimates of the intervention effects on PTSS at T2 and T3 (p values ≤ .001).  The intervention significantly reduced PTSS to nonclinical levels at T2 for boys but increased psychological distress in some children. Among girls, low peritraumatic dissociation predicted lower PTSS at T2 in the intervention group, while no effect was observed for those with medium or high peritraumatic dissociation. |
| Shoshani (2021) [13] | Israel | Quasi-experimental repeated-measures design | *N* = 2228 (51.1% female), *M_age_* = 13.06, age range 11-15  *N*_intervention_= 1120  *N*_waitinglist_ = 1108 | 1. Maytiv [“Doing Good”] program for fifteen 2-hour sessions, one every two weeks.  2. Waiting list | Trained teachers, supervised by clinical psychologists with masters level training | Exposure to political violence (PLE), mental health symptoms (BSI-18), satisfaction and happiness (SWLS), positive and negative affect (PANAS-C), attitudes toward the adversary and conflict resolution (APQ), peace attitude (PAS) | The intervention led to significant increase in well-being (F(1,36) = 30.07, p < 0.001, ηp² = 0.45) and reductions in generalized distress (F(1,36) = 37.95, p < 0.001, ηp² = 0.51), depression (F(1,36) = 40.83, p < 0.001, ηp² = 0.53), anxiety (F(1,36) = 49.33, p < 0.001, ηp² = 0.57), and PTSD (F(1,36) = 41.10, p < 0.001, ηp² = 0.53). There were also significant effects for social/instrumental support (F(2,36) = 8.45, p = 0.001, ηp² = 0.32), religion (F(2,36) = 5.75, p = 0.007, ηp² = 0.24), and self-distraction (F(2,36) = 4.94, p = 0.01, ηp² = 0.21).  Notably, in-person and hybrid participants reported greater social support and religious coping than those in remote sessions (p < 0.04). |
| Tol et al., 2008 [14] | Sulawesi, Indonesia | Cluster randomized trial | *N* = 403 (48.6% female), *M_age_* = 9.94, age range 7-15  *N*_intervention_ = 182  *N*_waitinglist_ = 221 | 1. Classroom-based intervention of 15 sessions, thrice per week over 5 weeks 2. Waiting list | Trained local ‘interventionists’ (>18 years with at least high school education); no details on supervision reported | Posttraumatic symptoms (CPSS), depressive complaints (DSRS), anxiety symptoms (SCARED-5), aggression (CAS-P), hope (CHS) | There were significant changes on means between the intervention and the waiting list groups on child-rated measures between baseline and first follow-up, but not between first and second follow-up. Changes generally remained at the second follow-up.  Significant treatment effect was found for PTSD symptoms (mean change: −2.78; 95% CI: 1.02 to 4.53) and hope (mean change: −2.21; 95% CI: −3.52 to −0.91), but not for depression, anxiety, or functional impairment. PTSD symptoms and functional impairment moderately improved in girls, while hope was retained in both boys and girls compared to the waitlisted group. For boys, no changes were found in traumatic idioms, depressive and anxiety symptoms, or functioning.  There were no significant differences on the parent-rated measures. |
| Tol et al. (2014) [15] | Burundi | Cluster randomized trial | *N* = 329 (48.0% female), age range 11-16  *N*_intervention_ = 153 *N*_waitinglist_ = 176 | 1. Classroom-based intervention of 15 sessions thrice per week over 5 weeks. 2. Waiting list | Trained local non-specialized facilitators (with at least high school education and one year of implementation supervision prior to the study) | PTSD symptoms (CPSS), depressive symptoms (Depression Self-Rating Scale) anxiety (SCARED), sense of hope (Children’s Hope Scale), coping (Kidcope), social support (SSIS) | There were no significant differences between the intervention and control group.  In the intervention group, children from larger households showed greater improvement in depression and functional impairment, while living with both parents was linked to reduction in PTSD and depression. In the control group, living with both parents was linked to increased depression. |
| Veronese & Barola (2018) [16] | Gaza Strip, Palestinian Territories | Randomized quasi-experimental design | *N* = 64  *N*_intervention_ = 28 (25% female), *M*_age_ =11.33, age range 8-12  N_waitinglist_ = 36 (28% female) *M*_age_ = 11.68, age range 9-13 | Narrative psychosocial intervention of 6 4-hour sessions | Teachers and mental health practitioners, supervised by international experts. A trained researcher administered scales | Life satisfaction (MSLSS); Face Scale (FS); optimism and pessimism (Y-LOT); affect (PANAS) | Children in the intervention group experienced increased life satisfaction from pre-test to T1. Specifically, this was found for greater appreciation for friends, family, themselves, and their living environment. There was also increased life satisfaction and happiness. (Effect sizes *d* = 0 .40-3.80). Younger children experienced more negative emotions than older, but no gender effect was found.  At T2, females scored higher than males on overall life satisfaction and satisfaction with friends, living environment, and self (all p < 0.05). No age effect was found. |
| **Civilians - Non-trauma-focused group intervention** | | | | | | | |
| D’Andrea et al. (2023) [17] | Gaza Strip, Palestinian Territories | Randomized controlled trial | *N*_intervention_ = 2341 (49.3% female), *M*_age_ = 10.70, age range 8-13  NB: the waiting list group is not described | 1. Eye to the Future intervention, lasting six months with 7.5 hours of program time each week across 3 afternoons  2. Waiting list | ‘Mentors’ trained by CARE staff, under supervision of a program coordinator on site | Mental Health (YSL; CBCL), Resilience (BRS), physical violence attitudes (NOBAGS), Social connections and loneliness (PNDLS) optimism (LOT-R) | There were statistically significant symptom reductions from the first three weeks of the intervention to the last three weeks of the intervention, across all symptom measures (Cohen’s d = .47 - .97). There were also significant reductions in children scoring above clinical thresholds at baseline versus at the end of the program (YSL: 61.3% versus 15.6%, CBCL: 78.6% versus 31.3%). |
| **IDPs - Non-trauma focused group interventions** | | | | | | | |
| Bolton et al. (2007) [18] | Uganda | Randomized controlled trial | *N* = 314 (57.3% female), *M*_age_ = 14.97, age range 14-17  *N*_psychotherapy_ = 105  *N*_creativeplay_ = 105  *N*_waitinglist_ = 104 | 1. Group interpersonal psychotherapy consisting of 16 weekly 1.5-2-hour sessions.  2. Creative play consisting of 16 weekly 1.5-2-hour sessions.  3. Waiting list | Local trained facilitator, supervised by World Vision Uganda staff with prior group interpersonal psychotherapy experience and War Child Holland psychosocial specialist | Depression, anxiety and conduct problems (APAI) | There were reduced depression symptoms post-intervention for all groups. The IPT-G group showed significantly greater decline than the waitlist group (adjusted mean difference of 9.79 points (95% CI: 1.66–17.93)), but the CP group showed a smaller, non-significant decline.  Recovery rates were 37.1% (IPT-G), 12.4% (CP), and 13.5% (waitlist), while remission rates were 29.1% (IPT-G), 6.7% (CP), and 8.9% (waitlist). |
| Thabet et al. (2005) [19] | Gaza Strip, Palestinian Territories | Quasi-randomized design | *N* = 111 (75.7% female), *M*_age_ = 12.33, age range 9-15  *N*_intervention_ = 47  *N*_education_ = 22  *N*_waitinglist_ = 42 | 1. Crisis intervention of 7 weekly sessions of unspecified duration.  2. Teacher education of 4 sessions of unspecified duration and frequency.  3. Waiting list | Crisis intervention by a child psychiatrist with a psychologist and social worker as facilitator, education by trained teachers | PTSD symptoms (UCLA PTSD index), depression (CDI) | The crisis intervention had no significant impact on PTSD or depression.  All three groups had similar baseline CPTSD-RI and CDI scores, with no significant changes over time—except a near-significant decrease in intrusion scores (p = 0.06) in the intervention group. ANOVA revealed no significant group differences for total PTSD, intrusion, avoidance, arousal, or depression scores. |

***General abbreviations:*** ACT = acceptance and commitment therapy; DSM = Diagnostic and Statistical Manual of Mental Disorders; PTSD = Post-Traumatic Stress Disorder; TF-CBT = Trauma-Focused Cognitive Behavioral Therapy.

***Questionnaire abbreviations:*** APQ = Adversary Perception Questionnaire; APAI = Acholi Psychosocial Assessment Instrument; BRS = Brief Resilience Scale; BSI-18 = Brief Symptom Inventory-18 items; CAS-P = Children’s Aggression Scale for Parents; CBCL = Child Behavior Checklist; CDI = Child Depression Inventory; CHS = Children’s Hope Scale; CLS = Children’s Loneliness Scale; CPSS = Child Posttraumatic Symptom Scale; cPTCI = Children’s Post-Traumatic Cognitions Inventory; CPTS-RI = Child Post Traumatic Stress Reaction Index; CRIES = Children's Revised Impact of Event Scale; CRIES-13 = Children’s Impact of Events Scale-13 items; CSQ-8 = Client Satisfaction Questionnaire 8 items; DISC = Diagnostic Interview Schedule for Children; DPS = Diagnostic Predictive Scales; DSRSC = Depression Self-Rating Scale for Children; ERQ = Emotion Regulation Questionnaire; FQS = Friendship Qualities Scale; IES-R = Revised Impact Event Scale; LOT-R = Life Orientation Test; MAQ = Multiple Aggression Questionnaire; MSC-SF = Mental Health Continuum-Short Form; MSLSS = Multidimensional Students’ Life Satisfaction Scale; NOBAGS = Normative Beliefs about Aggression Scale; PANAS-C = Positive and Negative Affect Schedule for Children; PAS = Peace Attitudes Scale; PDEQ = modified Peritraumatic Dissociative Experiences Questionnaire; PLE = Political Life Events Scale; PNDLS = Peer Network and Dyadic Loneliness Scale; SCARED = Screen for Child Anxiety Related Emotional Disorders; SCARED-5 = Self-Report for Anxiety Related Disorders 5-item version; SDQ = Strengths and Difficulties Questionnaire; SSIS = Social Support Inventory Scheme; SWLS = Satisfaction with Life Scale; Y-LOT = Youth Life Orientation Test; YSL = Youth Self Report

**Supplementary Table 2: MHPSS interventions targeting children, adolescents, and adults**

| **Study** | **Location** | **Design** | **Participants** | **Intervention and control** | **Provider and supervision** | **Targets** | **Outcomes** |
| --- | --- | --- | --- | --- | --- | --- | --- |
| **Civilians - Trauma-focused intervention** | | | | | | | |
| Schmitt et al. (2022) [20] | Democratic Republic of Congo | Uncontrolled | *N* = 200 (54% female), *M*_age_ = 35, age range 16-93  NB: no control group | 1. NETfacts consisting of 7 individual NET trauma therapy sessions and 4 NETfacts community-based sessions in 20 days. NETfacts session duration was 60-90minutes, no duration reported for individual NET sessions. | Licensed clinical psychologist and trained local counsellors (Congolese nurses, community members and not-for-profit organization members) supervised by clinical psychologists and experienced local counsellors | PTSD (PSS-I-5); depression (PHQ-9); social measures (SoRS; IRMA; ABSV; SAQ) | Participants with > 18 traumatic events showed a flattened curve of the building block effect at post follow-up. Experiencing at least one new traumatic event since baseline was associated with a higher PSS-I sum score (ß = .22, SE = .11, z = 2.05, p = 0.041).  Participants with a high number of traumatic events showed decreased sum scores of rape myth acceptance at post follow-up.  There was also an increase in affirmations of the item “willingness to support a family members affected by sexual violence” (t(159) = 3.43, p = 0.001, N = 200) |
| **Civilians – Non-trauma-focused interventions** | | | | | | | |
| Altawil et al. (2018) [21] | Gaza Strip, Palestinian Territories | Non-randomized experimental design | *N* = 227 (79.9% female), *M*_age_ = not reported, age range 12-75 years  *N*_familytherapy_ = 80  *N*_psychosocial_ = 90  *N*_communitywellness_ = 115 | 1. Family Therapy consisting of 12 sessions, once per week.  2. Psycho-social support of 6 sessions.  3. Community wellness focusing consisting of 12 sessions, over 3 months. | Clinical psychologists, psychiatrists, social and family workers, and activity facilitators with PTSD experience, using written intervention protocols; no details on supervision reported | PTSD symptoms (PTSD-SRII) | There was significant reduction in PTSD symptoms from pre- to post-intervention **(**t(282) = 38.41, p < 0.01) for all three interventions and across all PTSD subscales.   Family therapy was most effective in reducing obsessive-compulsive symptoms, community wellness focusing in avoidance symptoms, negative changes, and irritability symptoms, and psycho-social support in personal/professional functional accomplishments. |
| Ayoughi et al. (2012) [22] | Afghanistan | Randomized clinical trial | *N* = 66 (95% female), *M_age_* = 33.2, age range 14-60  *N*_intervention_ = 31  *N*_routine_ = 35 | 1. Psychosocial counselling consisting of 5-8 individual sessions.  2. Routine medical treatment (medication), no details on intervention duration reported | Experienced local physicians; no details on supervision reported | Depression and anxiety (HSCL) | There was a significant interaction of treatment x time in depression score (F(1,59) = 175.21, p < .001). At 3-month follow-up, the psychosocial counselling group showed significantly lower depression scores than the medication group (*p* < .001).  Anxiety scores significantly decreased in the counselling group, but not in the medication group (p < .001). |
| Carter et al. (2023) [23] | Iraq | Single arm pilot study, uncontrolled | Caretakers:  *N* = 8 (100% female), *M*_age_ = 42, age range 25 - 70  Children: *N* = 8 (0% female), *M*_age_ = 9, age range 8-12  NB: no control group | 1. Caregiver group intervention consisting of 6 weekly 2-hour sessions | Two group facilitators supervised by an MSF clinical supervisor | Child emotional and behavioural difficulties and positive attributes (SDQ-P), Child trauma-related mental health difficulties (CRIES-13), Child psychosocial functioning (CORS), Caregiver mental health (SRQ-20), Parental self-efficacy (BPSES) | There were large reductions (*d* > 0.8) in child emotional and behavioural difficulties, child trauma-related mental health difficulties, caregiver mental health and improvements in child prosocial behaviours and parental self-efficacy following the caregiver group intervention. |
| Gaboulaud et al. (2010) [24] | Gaza Strip and West Bank, Palestinian Territories | Uncontrolled | *N* = 1773 (47.7% female), *M*_age_ = 20, age range 0-78  *N*_individualtherapy_ = 887  *N*_familytherapy_ = 709  *N_dyadtherapy_* = 177 | 1. Individual psychotherapy  2. Family therapy  3. Caregiver/child dyad therapy  All following the MSF programme, with approximately 6 sessions (range 1-52) over a median of 11 weeks (range 6-19). | Expatriate therapists in the field with monthly supervision with expert psychiatrist | PTSD, complex PTSD and anxiety disorders (culturally specific modified DSM-IV classifications), and psychological distress (Global assessment of functioning) | Therapists reported  that at the last session, 80 % (1211/1514) of the patients improved with therapy. This was not significantly different for male versus female participants (*p* = 0.06) or different age groups (*p* = 0.09). |
| Hewitt-Ramírez et al. (2020) [25] | Colombia | Quasi-experimental design | *N* = 966  *N*_children_ = 127 (42.5% female), *M*_age_ = 7.6 (*SD* 2.5, range reported) *N*_adolescents_ = 162 (61.1% female), *M*age 14.6 (*SD* 1.2, no range reported)  *N*_adults_ = 677 (65.3% female), *M*age = 43.4 (*SD* = 13.4, no range reported) | 1. Primary care mental health Well-Being Program, consisting of nine, 2-hour sessions; no details surrounding intervention duration reported | Psychologists and psychology students (final year of studies); no details on supervision reported | Children:  Internalizing and externalizing behaviours & eight specific syndromes (CBCL); post-traumatic distress (TSCC); and resilience (ERE)  Adolescents:  Internalizing and externalizing behaviours & eight specific syndromes (YSR); coping strategies (KIDCOPE) and resilience (ERE)  Adults:  Depression and anxiety (SRQ); Post-traumatic stress (TEPT alcohol use (CAGE); coping strategies (EEC-M); and resilience (RSA) | For children and adolescents, there was a small (*d* ≤ 0.20) and intermediate (0.20 < *d* ≤ 0.50) reduction in internalizing and externalizing behaviours, total number of problems, and specific syndromes. There was also a small and intermediate effect size in use of avoidant coping strategies, but no change in resilience.  For the adults, there was a small reduction in depression, anxiety, and alcohol use (*d* ≤ 0.20). There was also a significant decrease in PTSD and avoidant coping skills (both *d* ≤ 0.20). |
| Redlener et al. (2025) [26] | Ukraine | Uncontrolled | *N*_parents_ = 963; 100% female)  *M*_age_ = unknown  Nc_hildren_ = 1291 (49.0% female), *M*_age_ = 9.3, age range 6-16 years | 1. Age-appropriate 6-day psychosocial recovery camp using a mother-child intervention model, based various play, art, yoga and cognitive approaches | Recreational counselors and mental health professionals (Master’s level) under coordinated care and with ongoing psychotherapy training | Posttraumatic stress symptoms (CATS-2), unstructured exit interviews with mothers discussing concerns and observed changes | 75.0% of the children had an overall improvement in psychosocial state after the intervention, including being more able to enjoy activities, being more physical active, having better communication, feeling less isolated, and experiencing improved sleep.  Results for the adult group are not reported. |
| Somer et al. (2005) [27] | Israel | Quasi-experimental controlled pilot study | *N* = 31 (85% female), *M*age not reported, age range not reported (*N* < 18 = 2 vs 2)   *N*_phoneCBT_ = 17  *N*standard = 14 | 1. Cognitive-behavioral therapy delivered via phone; no details on duration reported  2. Standard (hotline) care; no details on duration reported | Hotline volunteers who received CBT training; no details on supervision reported | Anxiety (STAI and SUD), and worrying (MAWI) | The CBT group had a significantly larger decrease in mean SUDS scores (controlled for baseline score) than the control group (*R^2^* = 0.24-0.48), STAI scores (*R^2^* = 0.21-0.56), and MAWI scores (*R^2^* = 0.26-0.48). |
| **IDPs – Trauma-focused interventions** | | | | | | | |
| Ertl et al. (2011) [28] | Uganda | Randomized controlled trial | *N* = 85, (55.3% female), *M_age_* = 18, age range 12-25  *N*_NET_ *= 29, N*_academiccounselling_ *= 28,*  *N*_waitinglist_ *= 28* | 1. NET consisting of 8 sessions of 90-120 minutes, 3 times per week.  2. Academic counselling of 8 sessions of 90-120 minutes, 3 times per week.  3. Waiting list | Local lay counsellors with supervision for case discussion and treatment (video) recording | PTSD diagnosis and symptom severity (CAPS-IV-R), depression and suicide risk (MINI), and stigma (shortened PSQ) | PTSD symptom severity showed a mean change difference between pre-treatment and 12-month follow-up of -14.06 when comparing NET with academic counselling, and -13.04 when comparing NET with waiting list.  The time x treatment interaction was not significant in a mixed-effects model for PTSD severity (*p* = 0.24), depression, suicidal ideation, and stigmatization, but significant for functioning impairment and guilt.  The within-treatment effect size on PTSD scores was largest for NET (d=1.80), with a 51.6% symptom score reduction. |
| **IDPs – Non-trauma-focused interventions** | | | | | | | |
| Martínez Torre et al. (2022) [29] | Nigeria | Retrospective analysis, uncontrolled | *N* = 6046 (78% female), *M*_age_ = 32.7 (*SD* = 13.1)  NB: no control group | MHPSS services provided by MSF; on average 1.8 individual sessions (*M* = 2)); no details regarding intervention duration reported | Community workers, lay counselors , medical doctors and clinical psychologists, supervised by clinical psychologists and psychiatrists | Mental health symptoms in general (MHGS scale and CGI-S) | As reported by the therapist, almost all patients (91.5%) improved ‘much’ or ‘very much’ following the treatment.  On the patient reported scale, 72.6% of the adults reported improvement, and only 1.1% (*n* = 7) reported worsening of their symptoms. For the children, only 45.2% reported improvement, though a similar number of 1.3% (*n* = 7) reported worsening.  Patients with posttraumatic or depressive symptoms were more likely to improve (*p* < 0.001) as were those with more severe symptoms. |
| Sonderegger et al. (2011) [30] | Uganda | Pilot evaluation with convenience sample | *N* = 202  *N*_intervention_ = 90 (40% female, M*age* = 30.69 (*SD* = 10.28)  *N*_waitinglist_ = 112 (75% female, *M*_age_ =27.86 years (*SD* = 14.14) | 1. Culturally sensitive CBT-based intervention (EMPOWER) of 13, 2-hour sessions; no details on intervention duration reported.  2. Waiting list | Local Acholi national facilitators trained and supervised by licensed clinical psychologist | Depressive- and anxiety-like symptoms, and prosocial behaviours (APAI) | There were significant differences between the treatment and control groups at post-assessment (*p* < .001) and 3-months follow-up (*p* < .001). There were also significant time x treatment effects for the depressive- and anxiety-like symptoms subscales (*p* < .001) and the prosocial scale (*p* < .01). |
| **Civilians and IDPs – Non-trauma-focused interventions** | | | | | | | |
| Andersen et al. (2020) [31] | Burundi, Central African Republic, Democratic Republic of the Congo, Mali, Nigeria and South Sudan | Non-controlled review | *N* = 5527 (81.24% female; 79% civilians; 21% migrants), age range 2-81 (11.47% younger than 18)  *N*_intervention_ = 5527 | 1. Unstructured psychological treatment of variable duration, with up to 10 weekly sessions for individual sessions, and up to 3 or more for group sessions. | Local counselors without formal psychological training, trained and supervised by ICRC MHPSS team | PTSD symptoms (IES-R), depression and anxiety (DASS-21), and daily life function (ICRC Functionality Scale for Africa) | Improvement of psychological distress (all measures combined) was mainly associated with having high distress at baseline (aOR = 28.70, *p* < 0.0001). There was a linear trend between likelihood of improved distress and number of sessions.  For the PTSD symptoms, higher scores at baseline were associated with more improvement (aOR 33.70, p ≤ 0.0001), as were low functioning and lacking social support. |
| Andersen et al. (2022) [32] | Democratic Republic of Congo, Mali and Nigeria | Uncontrolled retrospective cohort study | *N* = 6413 (80.31% female; 27% IDPs), age range 0-81 years (no *M*_age_ reported; 8.67% younger than 18) | 1. Individual psychological support (3-4 sessions over 15-60 days) and group psychological support (Msession = 7) following the ICRC MHPSS framework | Lay counsellors trained and supervised by ICRC MHPSS team | PTSD symptoms (IES-R), Depression and anxiety (DASS-21), Daily life function (ICRC Functionality Scale) | At post-assessments, PTSD symptoms improved among 92.70% of patients (95% CI −36.12; −33.69), depression and anxiety symptoms improved among 96.58% of patients (95% CI −37.64; −35.79), and ICRC functioning scale improved among 82.26% of patients (95% CI 4.20; 4.60). |
| Sanchez-Padilla et al. (2009) [33] | Colombia | Uncontrolled | *N* = 2411 (65.6% female in urban area; 68.6% female in rural areas; % IDPs not reported)  Age = 83.3% ≥ 15 years in urban area; 70.8% ≥ 15 years in rural areas  *N*_individual_ = 2054  *N*_group_ = 213  *N*_dyad_ = 103 | 1. Psychotherapy (individual, group or dyad) following the MSF Mental Health Program. Urban area: open schedule sessions in outpatient clinic; no details regarding intervention duration reported. Rural areas: fixed 5 session schedule via mobile clinics; no details on intervention duration reported. | Licensed psychologist; no details on supervision reported | Distress and psychological symptoms (DSM-IV criteria), daily activities, personal abilities, and capacity for problem-solving. | Following the psychotherapy, 89% of the children was reported as improved and 91% of the adults.  There is no separate reporting for the type of treatment received. |

***General abbreviations:*** DSM = diagnostic and statistical manual of mental disorders; ICRC = International Committee of the Red Cross; MHPSS = Mental Health and Psychosocial Support; MSF = Médecins Sans Frontière; NET = Narrative Exposure Therapy; PTSD = post-traumatic stress disorder; TF-CBT = trauma-focused cognitive behavioral therapy.

***Questionnaire abbreviations:*** ABSV = Attitudes and Beliefs towards Survivors of Sexual Violence Scale; APAI = Acholi Psychosocial Assessment Instrument; BPSES = The Brief Parental Self-Efficacy Scale; CAGE = Cute-Down, Annoyed, Guilty, Eye-Opener; CAPS-IV = Clinician-Administered PTSD Scale for DSM-IV; CATS-2 = Child and Adolescent Trauma Screen 2; CBCL = Child Behavior Checklist; CGI-S = Clinical Global Impression – Severity; CORS = Child Outcome Rating Scale; CRIES-13 = Children's Revised Impact of Event Scale – 13 items; DASS-21 = Depression, Anxiety and Stress Scale - 21 Items; EEC-M = Modified Scale of Coping Strategies for Adults; ERE = Resilience Scale for School-Aged Children; HSCL = Hopkins Symptom Checklist; IES-R = Revised Impact Event Scale; IRMA = Illinois Rape Myths Acceptance Scale; KIDCOPE = Scale of Coping Strategies for Adolescents; MHGS = Mental Health Global State; MINI = Mini International Neuropsychiatric Interview; PHQ-9 = Patient Health Questionnaire – 9 items; PSQ = Perceived Stigmatization Questionnaire; PSS-I-5 = PTSD Symptom Scale-Interview for DSM-5; PTSD-SRII = Posttraumatic Stress Disorder Self-Report Inventory; RSA = Resilience Scale for Adults; SAQ = Social Acknowledgement Questionnaire; SDQ-PF = Strengths and Difficulties Questionnaire - Parent Form; SoRS = Social Reconstruction Scale; SRQ-20 = The Self Reporting Questionnaire – 20 items; STAI = State-Trait Anxiety Inventory; SUD = Subjective Unit of Distress; TEPT = Posttraumatic Stress Test for Adults; TSCC = Trauma Symptom Checklist for Children; YSR = Youth Self Report

**Supplementary Table 3: MHPSS interventions targeting adults**

| **Study** | **Location** | **Design** | **Participants** | **Intervention and control** | **Provider and supervision** | **Targets** | **Outcomes** |
| --- | --- | --- | --- | --- | --- | --- | --- |
| **Civilians - Trauma-focused interventions** | | | | | | | |
| Bonilla-Escobar et al. (2018) [34] | Colombia | Randomized controlled trial | *N* = 346 (86.4% female), *M_age_* = 43.0 (*SD* = 16.6)   *N*_intervention_ = 175 *N*_waitinglist_ = 171 | 1. Common Elements Treatment Approach (CETA) consisting of 8-12 individual sessions; no details surrounding intervention duration reported 2. Waiting list | Lay psychosocial community workers, supervised by a clinical psychologist | General mental health (TMHS); PTSD (PCL-C; HTQ), and depression and anxiety (HSCL-25) | There was a significant improvement on symptom scores (general mental health, PTSD, depression and anxiety) following CETA compared to the waitlist condition in one of the study locations, but not in the other (where the number of attended sessions was lower (*p* = 0.006). |
| Knaevelsrud et al. (2015) [35] | Iraq | Randomized controlled trial | *N* = 159 (71.7% female), *M_age_* = 28.1 (*SD* = 7.43)  *N*_intervention_ = 79  *N*_waitinglist_ = 80 | 1. Culturally adapted Internet-based CBT of 2 weekly 45-minute sessions over 5 weeks 2. Waiting list | Native Arabic-speaking psychotherapists or psychiatrist, with professional supervision | PTSD (PDS), other mental health symptoms (HSCL-25; SCL-90-R), and quality of life (EUROHIS-QOL) | There was significant improvement in all symptom scores following the CBT, with a large reduction in PTSD symptoms (*d* = .72) and large effect on the other outcomes (*d* = .56-1.03). There was no significant change in the waiting list group. |
| Robjant et al. (2022) [36] | Democratic Republic of Congo | Prospective randomized controlled trial | *N* = 1066 (50% female), *M_age_* = 36.3 (*SD* = 16.7)  *N*_NETfacts_ = 493  *N*_NET_ = 573 | 1. NETfacts consisting of individual NET sessions and NETfacts community-based sessions, no duration or number of sessions reported.  2. Individual NET therapy, no duration or number of sessions reported. | Local trained trauma counselor, supervised by clinical psychologists | Rape myth acceptance and stigmatization (IRMA), and victimization and perpetration (ATSS) | Compared to the NET only group, NETfacts led to an increased reduction in acceptance of rape myth and an associated reduction in actual victimization and perpetration as well as an immediate reduction in PTSD and increased likelihood of engagement in NET subsequently.  While there was no evidence for a direct effect of NETfacts on victimization and perpetration, the mediation effect of treatment through rape myth acceptance on social (z = –2.56, P = 0.010) and physical victimization (z = –2.2, P = 0.027) and perpetration (z = –6, P < 0.001) was significant at 6-mo follow-up. |
| Wagner et al. (2012) [37] | Iraq | Uncontrolled | *N* =15 (86.7% female), *M*_age_ = 29.3 (*SD* = 7.1)  *N*_intervention_ = 15  NB: no control group | 1. Culturally adapted Internet-based CBT consisting of 2 weekly 45-minute assignments over 5 weeks | Native Arabic-speaking psychotherapists or psychiatrists, with professional supervision | PTSD (PDS), other mental health symptoms (HSCL-25) and quality of life (EUROHIS-QOL) | The intervention led to highly significant decrease of total PTSD (*d* = 1.57), and the intrusions, avoidance and hyperarousal subscales (*d* = 1.23-1.44). There was also a significant reduction of depression and anxiety (*d* = 1.50-1.51) and a significant increase of quality of life (*d* = 1.17). |
| Zemestani et al. (2022) [38] | Iraq | Pilot randomized controlled trial | *N* = 48 (100% female), *M_age_* = 32.91 (*SD* = 5.3)  *N*_intervention_ = 24  *N*_waitinglist_ = 24 | 1. Culturally adapted trauma-focused CBT of 12 individual sessions over 12 weeks 2. Waiting list | Master’s-level clinical psychologists with at least 6 years of professional experience, supervised by certified psychologist | PTSD (PCL-5), other mental health symptoms (DASS-21, DERS), and quality of life (WHOQOL-BREF) | The TF-CBT group showed a significantly larger decrease in PTSD symptoms (*g* = 1.30, *p* < 0.001), depression (*g* = 1.19), anxiety (*g* = 1.19) and stress (*g* = 0.97) than the waiting list group. They also showed a larger decrease in emotion regulation difficulties (*g* = 1.35) and larger increase in quality of life. These changes were all significant for the TF-CBT group (*p* all < 0.001), but not for the waiting list group. |
| (Zoellner et al. (2021) [39] | Somalia | Pre-post feasibility uncontrolled trial | *N* = 26 (54% female) *M_age_* = 27.62 (*SD* = 7.35)  *N*_intervention_ = 26  NB: no control group | 1. Islamic Trauma Healing Intervention consisting of 6 group sessions; no details surrounding intervention duration reported | Lay leaders, supervised by clinical psychologists | PTSD (PDS-5), other mental health symptoms (PHQ-9; SSS-8), and quality of life (WHO-5) | There were large effects of the intervention on PTSD severity (*g* = 1.91), depression, somatic symptoms, and well-being (*g* = 1.31-2.74). Notably, before the intervention 80.8% of participants met PTSD diagnosis, and at post-intervention this dropped to 15.4%. Higher pre-treatment severity was associated with larger improvement. |
| **Civilians – Non-trauma-focused interventions** | | | | | | | |
| Castro-Camacho et al. (2023) [40] | Colombia | Randomized clinical trial | *N* = 200 (80% female) *M_age_* = 43.1 (*SD* = 11.9)  *N*_intervention_ = 120  *N*_waitinglist_ *= 80* | 1. Contextual Adaptation of the Unified Protocol in Multiple Emotional Disorders, consisting of 12-14 individual sessions in 7 weeks.  2. Waiting list | Graduate clinical psychology students, supervised by principal investigator | PTSD (PCl-5), and anxiety, depression and somatic symptoms (PHQ-9) | The treatment group showed significant and large reductions in  in PTSD symptoms (*d* = .90), anxiety (*d* = .82), depression (*d* = .77) and somatic symptoms (*d* = .75). The results were similar at follow-up, indicating lasting results of the intervention at least 3-months after. |
| Lahutina et al. (2024) [41] | Ukraine | Cross-sectional observational study | *N = 3740* (unknown % female), *M*_age_ = 29.00 (SD = 9.13)  NB: no control group | 1. Psychological first aid (PFA) digital intervention using the PFA chatbot “Friend” | Delivered via Telegram Messenger; follow-up referral to local licensed psychologist | Open questions that assessed predictors of severity of perceived stress levels: namely parenthood (having children), feelings of insecurity, and loneliness | There was a medium to large decrease in stress levels (d = .73) following the PFA chatbot intervention. Higher stress levels were predicted by having children (parenthood; F = 5.54), feelings of safety (F = 28.84), and feelings of Loneliness (F = 32.99). |
| Rattner et al. (2023) [42] | Colombia | Uncontrolled, mixed-explanatory sequential design | *N* = 39 (58% female) *M_age_* = 24.47 (*SD* = 5.59)  *N*_intervention_ = 39  NB: no control group | 1. Community-based psychosocial support (CB-PSS) consisting of 8 group sessions in 8 weeks. | Community psychosocial agents, supervised by psychologist or social worker | Mental health symptoms, (PCL-C, Hopkins Symptom Checklist), and wellbeing (WHO-DAS) | The intervention led to a significant increase in wellbeing (F = 30.07, *p* < 0.001, eta^2^ = 0.45) and decreased generalized distress, depression, anxiety, and PTSD (eta^2^ = 0.51-0.57). There were no significant differences regarding functional impairment or community efficacy. |
| **Veterans or ex-combatants - Trauma-focused interventions** | | | | | | | |
| Köbach et al. (2015) [43] | Democratic Republic of Congo | Semi-randomized controlled trial | *N* = 98 (0% female) *M_age_* = 23.48 (*SD* = 5.81)   *N*_FORNET_ = 49  *N*_TAU_ = 49 | 1. Adapted NET (FORNET) of 5 individual sessions and 2 group sessions; no details on intervention duration reported  2. Treatment-as-usual (medical care and psycho-social support/counselling) for 2-15 hours total | Local counselors, supervised by clinical experts (psychologists) | PTSD (PSS-I), aggression (AFAS), depression (PHQ-9), and drug dependence (TCU drug screen) | There was a greater reduction in PTSD symptoms in the FORNET group compared to the TAU at 6-months post-treatment (d = 0.72, p < 0.001). The remission rate was also substantially lower in the FORNET group, with 66% out of the initial 29 participants matching PTSD criteria being in remission (compared to 43% for the TAU group).  There were no significant effects on aggression and drug dependence. |
| Koebach et al. (2021) [44] | Democratic Republic of Congo | Randomized controlled trial | *N* = 448 (0% female), *M_age_* = 33 years (S*D* = 9)  *N*_FORNET_ = 224  *N*_TAU_ = 224 | 1. Adapted NET (FORNET) of 6 individual and 6 group sessions in 6 weeks; no details on intervention duration reported.  2. Treatment-as-usual through different NGOs: occupational training courses, empathic listening, problem-solving, and practical support | Congolese personnel who received a three-week FORNET training, supervised by clinical psychologist and trained Congolese supervisors | Aggression and violence (AFAS), and PTSD (PSSI) | Aggression, posttraumatic stress, depression, and drug involvement decreased more strongly in the FORNET group than in the TAU group (*R^2^* = 0.04-0.72). Current violent behavior and solidarity to (para)military life also both decreased more with FORNET (*R^2^* = 0.31-0.46).  There were no significant differential effects for ex-child versus adult soldiers. |
| **Veterans or ex-combatants – Non-trauma-focused interventions** | | | | | | | |
| Bogdanov et al. (2021) [45] | Ukraine | Randomized controlled trial | *N* = 302 (60% female), *M_age_* = 39.02 (S*D* = 10.47)  *N*_CETA_ = 129  *N*_briefCETA_ = 117  *N*_waitinglist_ = 56 | 1. Standard Common Elements Treatment Approach (CETA) of 8-12 sessions, no duration reported.  2. Shortened CETA of 5 sessions, with no duration reported.  3. Waiting list | Providers with minimal mental health training, supervised by CETA supervisors and local psychiatrists | Depression, posttraumatic stress, generalized anxiety (MHAI), and functioning (WHO-DAS) | There was a large decrease in depressive, PTSD and anxiety symptoms and an increase in functioning for both the standard CETA (d = .60-1.06) and brief CETA  (d = .46-.62) compared to waitlist.  The standard CETA group reported fewer symptoms and less dysfunction at post-assessment than the brief CETA group with small to medium effect sizes for the different scores (*d* = 0.20-0.55). |
| Kukharuk et al. (2025) [46] | Ukraine | Randomized controlled trial (blocked randomization per age group) | *N* = 69 (0% female), *M*age not reported ( age range 24-45, with *n = 34*  Age: 24-30 years (n = 34); 31-40 years (n = 24); 40-45 years (n = 11)  N_videotherapy_ = 34  *N*_rehabilitation_ = 35 | 1. Immersive 360 degrees virtual  reality (video) therapy, consisting of 7 30-minute daily sessions.  2. Standard veteran rehabilitation program (psychotherapeutic), of unknown duration | Not specified | Anxiety and depression-related symptoms (HADS and MAEAS) | There was a small to medium reduction in anxiety (d = 0.43) and depression (d = 0.28) for the virtual reality group. This was significantly lower than in the standard rehabilitation control group (*p* = 0.027-0.039). |
| Trujillo et al. (2017) [47] | Colombia | Quasi-experimental design | *N* = 31 (6% female) *M_age_* = 37.16 (S*D* = 8.30)  *N*_socialcognitive_ = 16  *N*_reintegration_ = 15 | 1. Social Cognitive Training (SCT) consisting of 12 sessions in 14 weeks.  2. Conventional reintegration group of a 45min weekly session | Psychologist with advanced clinical training and expertise; no details on supervision reported | Emotion processing (ERT), aggression (ISCA), motives for aggression (IMA), interpersonal reactivity (IRI), and social skills (SS) | The SCT group showed improved recognition of neutral faces (*d* = .85), but no differences in aggression and social skills apart from a decrease in motives for aggression (eta2 = .57) following the intervention. These improvements were not found for the conventional reintegration group. However, no other significant differences were found after treatment. |

***General abbreviations:*** DSM = Diagnostic and Statistical Manual of Mental Disorders; FORNET = Narrative Exposure Therapy for Forensic Offender Rehabilitation; NET = Narrative Exposure Therapy; NGO = Non-Governmental Organization; PTSD = Post-Traumatic Stress Disorder; TF-CBT = Trauma-Focused Cognitive Behavioral Therapy; QOL = Quality of Life

***Questionnaire abbreviations:*** AFAS = Alcohol-Related Problems and Associated Factors Scale; ATSS = Attitudes Toward Sexuality Scale; DERS = Difficulties in Emotion Regulation Scale; ERT = Emotion Recognition Task; HADS = Hospital Anxiety and Depression Scale; HSCL-25 = Hopkins Symptom Checklist – 25 items; HTQ = Harvard Trauma Questionnaire; IMA = Motives for Aggression Inventory [Spanish acronym]; IRI = Interpersonal Reactivity Index; IRMA = Illinois Rape Myth Acceptance Scale; ISCA = Situation and Aggressive Behavior Inventory [Spanish acronym]; MAEAS = Mood and Anxiety Express Assessment Scale; MHAI = Mental Health Assessment Inventory; PCL-5 = PTSD Checklist for DSM-5; PCL-C = PTSD Checklist Civilian Version; PDS = Posttraumatic Diagnostic Scale; PHQ-9 = Patient Health Questionnaire – 9 items; PSS-I = PTSD Symptom Scale – Interview; SCL-90-R = Symptom Checklist-90-Revised; SS = Social Skills Scale; TCU drug screen = Texas Christian University Drug Screen; TMHS = Total Mental Health Symptoms Scale; WHO = World Health Organization, WHOQOL-BREF = WHO Quality of Life Scale; WHO-5 = World Health Organization-Five Well-Being Index; WHO-DAS = World Health Organization Disability Assessment

**References**

[1] S. J. Ahmadi, A. Tavoli, Z. Musavi, and J. Dainer-Best, “Acceptance and commitment therapy versus trauma-focused cognitive behavior therapy: A comparative study of the effects on the posttraumatic stress symptoms of female Afghan adolescents,” *Am. Psychol.*, vol. 79, no. 9, pp. 1452–1459, Dec. 2024, doi: 10.1037/amp0001451.

[2] P. O’Callaghan, J. McMullen, C. Shannon, and H. Rafferty, “Comparing a trauma focused and non trauma focused intervention with war affected Congolese youth: A preliminary randomised trial,” *Interv. J. Ment. Health Psychosoc. Support Confl. Affect. Areas*, vol. 13, no. 1, pp. 28–44, Mar. 2015, doi: 10.1097/WTF.0000000000000054.

[3] R. Berger, R. Pat-Horenczyk, and M. Gelkopf, “School-based intervention for prevention and treatment of elementary-students’ terror-related distress in Israel: A quasi-randomized controlled trial,” *J. Trauma. Stress*, vol. 20, no. 4, pp. 541–551, Aug. 2007, doi: 10.1002/jts.20225.

[4] R. Berger, M. Gelkopf, and Y. Heineberg, “A teacher-delivered intervention for adolescents exposed to ongoing and intense traumatic war-related stress: A quasi-randomized controlled study,” *J. Adolesc. Health*, vol. 51, no. 5, pp. 453–461, Nov. 2012, doi: 10.1016/j.jadohealth.2012.02.011.

[5] M. Diab, R. Punamäki, E. Palosaari, and S. R. Qouta, “Can psychosocial intervention improve peer and sibling relations among war‐affected children? Impact and mediating analyses in a randomized controlled trial,” *Soc. Dev.*, vol. 23, no. 2, pp. 215–231, May 2014, doi: 10.1111/sode.12052.

[6] M. Diab, K. Peltonen, S. R. Qouta, E. Palosaari, and R.-L. Punamäki, “Effectiveness of psychosocial intervention enhancing resilience among war-affected children and the moderating role of family factors,” *Child Abuse Negl.*, vol. 40, pp. 24–35, Feb. 2015, doi: 10.1016/j.chiabu.2014.12.002.

[7] E. Eiling, M. Van Diggele-Holtland, T. Van Yperen, and F. Boer, “Psychosocial support for children in the Republic of South Sudan: An evaluation outcome,” *Interv. J. Ment. Health Psychosoc. Support Confl. Affect. Areas*, vol. 12, no. 1, pp. 61–75, Mar. 2014, doi: 10.1097/WTF.0000000000000023.

[8] J. T. Forsberg and J.-H. Schultz, “Educational and psychosocial support for conflict-affected youths: The effectiveness of a school-based intervention targeting academic underachievement,” *Int. J. Sch. Educ. Psychol.*, vol. 11, no. 2, pp. 145–166, 2023, doi: 10.1080/21683603.2022.2043209.

[9] S. Kangaslampi, R. Punamäki, S. Qouta, M. Diab, and K. Peltonen, “Psychosocial group intervention among war‐affected children: An analysis of changes in posttraumatic cognitions,” *J. Trauma. Stress*, vol. 29, no. 6, pp. 546–555, Dec. 2016, doi: 10.1002/jts.22149.

[10] K. Peltonen, S. Qouta, E. El Sarraj, and R.-L. Punamäki, “Effectiveness of school-based intervention in enhancing mental health and social functioning among war-affected children,” *Traumatology*, vol. 18, no. 4, pp. 37–46, Dec. 2012, doi: 10.1177/1534765612437380.

[11] R.-L. Punamäki, K. Peltonen, M. Diab, and S. R. Qouta, “Psychosocial interventions and emotion regulation among war-affected children: Randomized control trial effects,” *Traumatology*, vol. 20, no. 4, pp. 241–252, Dec. 2014, doi: 10.1037/h0099856.

[12] S. R. Qouta, E. Palosaari, M. Diab, and R. Punamäki, “Intervention effectiveness among war‐affected children: A cluster randomized controlled trial on improving mental health,” *J. Trauma. Stress*, vol. 25, no. 3, pp. 288–298, Jun. 2012, doi: 10.1002/jts.21707.

[13] A. Shoshani, “Transcending the reality of war and conflict: Effects of a positive psychology school-based program on adolescents’ mental health, compassion and hopes for peace,” *J. Posit. Psychol.*, vol. 16, no. 4, pp. 465–480, Jul. 2021, doi: 10.1080/17439760.2020.1752778.

[14] W. A. Tol, I. H. Komproe, D. Susanty, M. J. D. Jordans, R. D. Macy, and J. T. V. M. De Jong, “School-based mental health intervention for children affected by political violence in Indonesia: A cluster randomized trial,” *JAMA J. Am. Med. Assoc.*, vol. 300, no. 6, pp. 655–662, Aug. 2008, doi: 10.1001/jama.300.6.655.

[15] W. A. Tol *et al.*, “School-based mental health intervention for children in war-affected Burundi: a cluster randomized trial.,” *BMC Med.*, vol. 12, p. 56, Apr. 2014, doi: 10.1186/1741-7015-12-56.

[16] G. Veronese and G. Barola, “Healing stories: An expressive-narrative intervention for strengthening resilience and survival skills in school-aged child victims of war and political violence in the Gaza Strip,” *Clin. Child Psychol. Psychiatry*, vol. 23, no. 2, pp. 311–332, Apr. 2018, doi: 10.1177/1359104518755220.

[17] W. D’Andrea *et al.*, “Growing Up on the Edge: A Community-Based Mental Health Intervention for Children in Gaza,” *Res. Child Adolesc. Psychopathol.*, vol. 52, no. 5, pp. 833–848, 2023, doi: 10.1007/s10802-023-01124-2.

[18] P. Bolton *et al.*, “Interventions for depression symptoms among adolescent survivors of war and displacement in Northern Uganda: A randomized controlled trial,” *JAMA J. Am. Med. Assoc.*, vol. 298, no. 5, pp. 519–527, Aug. 2007, doi: 10.1001/jama.298.5.519.

[19] A. A. Thabet, P. Vostanis, and K. Karim, “Group crisis intervention for children during ongoing war conflict,” *Eur. Child Adolesc. Psychiatry*, vol. 14, no. 5, pp. 262–269, 2005, doi: 10.1007/s00787-005-0466-7.

[20] S. Schmitt *et al.*, “Breaking the cycles of violence with narrative exposure: Development and feasibility of NETfacts, a community-based intervention for populations living under continuous threat,” *PLoS ONE*, vol. 17, no. 12 December, 2022, doi: 10.1371/journal.pone.0275421.

[21] M. A. S. Altawil, A. El Asam, and A. Khadaroo, “The Effectiveness of Therapeutic and Psychological Intervention Programs in PTC-GAZA.,” *J. Child Adolesc. Trauma*, vol. 11, no. 4, pp. 473–486, Dec. 2018, doi: 10.1007/s40653-018-0213-0.

[22] S. Ayoughi, I. Missmahl, R. Weierstall, and T. Elbert, “Provision of mental health services in resource-poor settings: A randomised trial comparing counselling with routine medical treatment in North Afghanistan (Mazar-e-Sharif),” *BMC Psychiatry*, vol. 12, Feb. 2012, doi: 10.1186/1471-244X-12-14.

[23] S. Carter *et al.*, “The feasibility and acceptability of implementing and evaluating a caregiver group intervention to address child mental health: A pilot study in Iraq,” *J. Affect. Disord. Rep.*, vol. 12, 2023, doi: 10.1016/j.jadr.2023.100503.

[24] V. Gaboulaud *et al.*, “Psychological support Palestinian children and adults: An analysis of data from people referred to the Médecins San Frontières programme for behavioural and emotional disorders in the occupied Palestinian territory,” *Interv. Int. J. Ment. Health Psychosoc. Work Couns. Areas Armed Confl.*, vol. 8, no. 2, pp. 131–142, Jul. 2010, doi: 10.1097/WTF.0b013e32833c1f8f.

[25] N. Hewitt-Ramírez, F. Juárez, A. J. Parada-Baños, X. Nuñez-Estupiñán, and L. Quintero-Barrera, “Efficacy of a primary care mental health program for victims of the armed conflict in Colombia,” *Peace Confl. J. Peace Psychol.*, vol. 26, no. 1, pp. 62–77, Feb. 2020, doi: 10.1037/pac0000436.

[26] I. Redlener, K. Dudashvili, O. Viontsek, R. Grant, and E. Roca, “Intervention for Psychological Trauma in Children Impacted by War in Ukraine,” *JAMA Netw. Open*, vol. 8, no. 3, p. e253057, Mar. 2025, doi: 10.1001/jamanetworkopen.2025.3057.

[27] E. Somer, E. Tamir, S. Maguen, and B. T. Litz, “Brief cognitive-behavioral phone-based intervention targeting anxiety about the threat of attack: A pilot study,” *Behav. Res. Ther.*, vol. 43, no. 5, pp. 669–679, May 2005, doi: 10.1016/j.brat.2004.05.006.

[28] V. Ertl, A. Pfeiffer, E. Schauer, T. Elbert, and F. Neuner, “Community-implemented trauma therapy for former child soldiers in Northern Uganda: A randomized controlled trial,” *JAMA*, vol. 306, no. 5, pp. 503–512, 2011, doi: 10.1001/jama.2011.1060.

[29] S. Martínez Torre *et al.*, “Severity, symptomatology, and treatment duration for mental health disorders: a retrospective analysis from a conflict-affected region of northern Nigeria.,” *Confl. Health*, vol. 16, no. 1, p. 41, Jul. 2022, doi: 10.1186/s13031-022-00473-x.

[30] R. Sonderegger, S. Rombouts, B. Ocen, and R. S. McKeever, “Trauma rehabilitation for war‐affected persons in northern Uganda: A pilot evaluation of the EMPOWER programme,” *Br. J. Clin. Psychol.*, vol. 50, no. 3, pp. 234–249, Sep. 2011, doi: 10.1348/014466510X511637.

[31] I. Andersen, R. Rossi, M. N. M. Yabutu, and I. Hubloue, “Integrating Mental Health and Psychosocial Support Into Health Facilities in Conflict Settings: A Retrospective Review From Six African Countries.,” *Front. Public Health*, vol. 8, p. 591369, 2020, doi: 10.3389/fpubh.2020.591369.

[32] I. Andersen, R. Rossi, and I. Hubloue, “Community-Level Mental Health and Psychosocial Support During Armed Conflict: A Cohort Study From the Democratic Republic of the Congo, Mali, and Nigeria.,” *Front. Public Health*, vol. 10, p. 815222, 2022, doi: 10.3389/fpubh.2022.815222.

[33] E. Sanchez-Padilla, G. Casas, R. F. Grais, S. Hustache, and M.-R. Moro, “The Colombian conflict: a description of a mental health program in the Department of Tolima.,” *Confl. Health*, vol. 3, p. 13, Dec. 2009, doi: 10.1186/1752-1505-3-13.

[34] F. J. Bonilla-Escobar *et al.*, “Randomized controlled trial of a transdiagnostic cognitive-behavioral intervention for Afro-descendants’ survivors of systemic violence in Colombia,” *PLoS ONE*, vol. 13, no. 12, Dec. 2018, doi: 10.1371/journal.pone.0208483.

[35] C. Knaevelsrud, J. Brand, A. Lange, J. Ruwaard, and B. Wagner, “Web-based psychotherapy for posttraumatic stress disorder in war-traumatized Arab patients: Randomized controlled trial,” *J. Med. Internet Res.*, vol. 17, no. 3, Mar. 2015, doi: 10.2196/jmir.3582.

[36] K. Robjant *et al.*, “NETfacts: An integrated intervention at the individual and collective level to treat communities affected by organized violence,” *Proc. Natl. Acad. Sci. U. S. A.*, vol. 119, no. 44, 2022, doi: 10.1073/pnas.2204698119.

[37] B. Wagner, W. Schulz, and C. Knaevelsrud, “Efficacy of an internet-based intervention for posttraumatic stress disorder in Iraq: A pilot study,” *Psychiatry Res.*, vol. 195, no. 1–2, pp. 85–88, Jan. 2012, doi: 10.1016/j.psychres.2011.07.026.

[38] M. Zemestani, A. F. Mohammed, A. A. Ismail, and A. A. Vujanovic, “A Pilot Randomized Clinical Trial of a Novel, Culturally Adapted, Trauma-Focused Cognitive-Behavioral Intervention for War-Related PTSD in Iraqi Women,” *Behav. Ther.*, vol. 53, no. 4, pp. 656–672, 2022, doi: 10.1016/j.beth.2022.01.009.

[39] L. A. Zoellner *et al.*, “Reaching the unreached: Bridging islam and science to treat the mental wounds of war,” *Front. Psychiatry*, vol. 12, Jun. 2021, doi: 10.3389/fpsyt.2021.599293.

[40] L. Castro-Camacho *et al.*, “Effects of a Contextual Adaptation of the Unified Protocol in Multiple Emotional Disorders in Individuals Exposed to Armed Conflict in Colombia: A Randomized Clinical Trial.,” *JAMA Psychiatry*, vol. 80, no. 10, pp. 991–999, Oct. 2023, doi: 10.1001/jamapsychiatry.2023.2392.

[41] S. Lahutina *et al.*, “A digital self-help tool to promote mental well-being for Ukrainians affected by war - Assessing predictors of stress,” *Neurosci. Appl.*, vol. 3, p. 104089, Jan. 2024, doi: 10.1016/j.nsa.2024.104089.

[42] M. Rattner *et al.*, “Piloting a community-based psychosocial group intervention designed to reduce distress among conflict-affected adults in Colombia: a mixed-method study of remote, hybrid, and in-person modalities during the COVID-19 pandemic.,” *Int. J. Ment. Health Syst.*, vol. 17, no. 1, p. 35, Oct. 2023, doi: 10.1186/s13033-023-00597-4.

[43] A. Köbach, S. Schaal, T. Hecker, and T. Elbert, “Psychotherapeutic Intervention in the Demobilization Process: Addressing Combat-related Mental Injuries with Narrative Exposure in a First and Second Dissemination Stage,” *Clin. Psychol. Psychother.*, vol. 24, no. 4, pp. 807–825, 2015, doi: 10.1002/cpp.1986.

[44] A. Koebach, S. Carleial, T. Elbert, S. Schmitt, and K. Robjant, “Treating trauma and aggression with narrative exposure therapy in former child and adult soldiers: A randomized controlled trial in Eastern DR Congo,” *J. Consult. Clin. Psychol.*, vol. 89, no. 3, pp. 143–155, 2021, doi: 10.1037/ccp0000632.

[45] S. Bogdanov *et al.*, “A randomized-controlled trial of community-based transdiagnostic psychotherapy for veterans and internally displaced persons in Ukraine,” *Glob. Ment. Health*, vol. 8, 2021, doi: 10.1017/gmh.2021.27.

[46] O. Kukharuk, Tkalich ,Kateryna, Kamash ,Nadia, and O. and Georgiou, “Effectiveness of immersive VR therapy in reducing stress-associated symptoms in Ukraine,” *Eur. J. Psychotraumatology*, vol. 16, no. 1, p. 2488097, Dec. 2025, doi: 10.1080/20008066.2025.2488097.

[47] S. Trujillo *et al.*, “Social cognitive training improves emotional processing and reduces aggressive attitudes in Ex-combatants,” *Front. Psychol.*, vol. 8, no. MAR, 2017, doi: 10.3389/fpsyg.2017.00510.
