# Supplementary material for: Mental health and psychosocial support interventions for populations affected by ongoing armed conflict: a scoping review
Source: BMJ Glob Health. 2026 May 27;11(5):e022708. doi: 10.1136/bmjgh-2025-022708 (PMC13218107; doi:10.1136/bmjgh-2025-022708)
Supplement: online supplemental file 2 [file bmjgh-11-5-s002.pdf]

BMJ Global Health Author Reflexivity Statement

Adapted from Morton, B., Vercueil, A., Masekela, R., Heinz, E., Reimer, L., Saleh, S., Kalinga, C., Seekles, M., Biccard, B., Chakaya, J., Abimbola, S., Obasi, A. and Oriyo, N. (2022), Consensus statement on measures to promote equitable authorship in the publication of research from international partnerships. Anaesthesia, 77: 264-276. https://doi.org/10.1111/anae.15597

**Study conceptualisation**	
1. How does this study address local research and policy priorities?	Georgia is a war-affected country, 20% of our territory is still occupied by Russian military forces who are regularly moving the current border deeper (so called "creeping occupation) keeping the border zone population under constant threat (see, e.g. the following international media coverage of creeping occupation: https://www.aljazeera.com/features/2022/7/3/the-georgian-village-facing-russian-creeping-occupation).<br>Stemming from the above, mental health and psychosocial support (MHPSS) responses amid ongoing armed conflict and ongoing threat represent a high priority in Georgia, both for research and MHPSS policy.
2. How were local researchers involved in study design?	The senior manuscript author, Dr Jana D. Javakhishvili (Georgia), was responsible for the conceptualization of the scoping review and provided senior leadership and guidance throughout the completion of the manuscript.
**Research management**	
3. How has funding been used to support the local research team(s)?	N/A
**Data acquisition and analysis**	
4. How are research staff who conducted data collection acknowledged?	Kindly see responses to question #2: Dr Jana D. Javakhishvili holds senior authorship on this publication
5. How have members of the research partnership been provided with access to study data?	All members involved in conceptualizing, identifying and reviewing articles for inclusion, drafting and revising the manuscript are acknowledged and included as co-authors. All co-authors have access to the study data (i.e., reviewed and included/excluded articles) through a shared document stored on, and accessible through, a shared drive.
6. How were data used to develop analytical skills within the partnership?	During the data analysis stage of the scoping review the international team jointly reflected on the data during regular meetings, which facilitated data analysis knowledge and skills sharing and contributed to enhancing analytical capacity within the partnership.
**Data interpretation**	
7. How have research partners collaborated in interpreting study data?	Multiple, recurring online meetings were held amongst all co-authors throughout the duration of the manuscript development to review and interpret scoping review findings.
**Drafting and revising for intellectual content**	

8. How were research partners supported to develop writing skills?	Multiple rounds of feedback were provided by senior authors and practitioners, including by Dr Jana D. Javakhishvili, throughout the drafting, and finalization, of the scoping review.
9. How will research products be shared to address local needs?	The research products (publication/s) will be presented for discussion with the local community of MHPSS professionals and decision- and policymakers.
**Authorship**	
10. How is the leadership, contribution and ownership of this work by LMIC researchers recognised within the authorship?	As mentioned above (question #2), the senior manuscript author, Dr Jana D. Javakhishvili (Georgia), was responsible for the conceptualization of the scoping review and provided senior leadership and guidance throughout the completion of the manuscript.
11. How have early career researchers across the partnership been included within the authorship team?	Early career researchers led the scoping review search, article screening and drafting of the manuscript. Early career researchers further hold shared first authorship of the manuscript.
12. How has gender balance been addressed within the authorship?	The authorship team reflects a balance in gender, geographic location, seniority, as well as content, disciplinary, and professional expertise (e.g., academic researchers, clinicians, policy, humanitarian and mental health experts).
**Training**	
13. How has the project contributed to training of LMIC researchers?	The paper, after it is published, will be included in the readings of the "MHPSS Responses to Catastrophes" course at Ilia State University MA program in Mental Health/Psychotraumatology.
**Infrastructure**	
14. How has the project contributed to improvements in local infrastructure?	To date, the project has not contributed to improvements in local infrastructure. However, following publication of the article, it will be presented and discussed with relevant stakeholders, decision- and policymakers.
**Governance**	
15. What safeguarding procedures were used to protect local study participants and researchers?	N/A
